# Highly Stretchable Polyrotaxane Thin‐Film Substrates for Printed Electronics

**DOI:** 10.1002/smsc.70412

**Published:** 2026-10-16

**Authors:** Vivek Manish, Roberto Baretta, Giada Bombardelli, Guglielmo Trentini, Chiara Morano, Andrea Gaiardo, Luisa Petti, Alvise Bagolini, Leonardo Pagnotta, Marco Frasconi, Maria F. Pantano

**Affiliations:** ^1^ Department of Civil Environmental and Mechanical Engineering University of Trento Trento Italy; ^2^ Department of Chemical Sciences University of Padova Padova Italy; ^3^ Center for Sensors and Devices Bruno Kessler Foundation Trento Italy; ^4^ Faculty of Engineering Free University of Bolzano‐Bozen Bolzano Italy; ^5^ Department of Mechanical Energy and Management Engineering University of Calabria Rende Cosenza Italy

**Keywords:** flexible sensors, freestanding films, mechanical properties, polyrotaxane, tensile test

## Abstract

Thin polymeric films with high mechanical robustness and stretchability are essential for the development of flexible and stretchable electronic devices. Here, we present a versatile strategy for fabricating freestanding polymer films with tunable mechanical properties while maintaining large stretchability. The films are fabricated by chemically cross‐linking multi‐arm poly(ethylene glycol) acrylate (4‐arm‐PEG‐A) with polyrotaxane (PR) units composed of α‐cyclodextrins (α‐CDs) threaded along a linear PEG backbone. By varying the degree of PR functionalization and its content, the mechanical properties of the films can be systematically tuned, enabling simultaneous enhancement of strength, toughness, and stiffness, with failure strains exceeding 150%. The resulting films were used as substrates for stretchable temperature sensors, which maintained stable performance under an applied strain of ~60%. These results demonstrate that polyrotaxane‐based thin films provide mechanically robust and functionally compatible substrates for stretchable electronics operating under large deformations.

## Graphical abstract

A versatile strategy is presented for tuning the mechanical properties of cross‐linked polymer films through the incorporation of polyrotaxanes into covalently cross‐linked multi‐arm PEG networks. Systematic control of polyrotaxane content and functionalization enhances strength, toughness, and stretchability, enabling freestanding substrates for stretchable temperature sensors that operate reliably under large mechanical deformation.

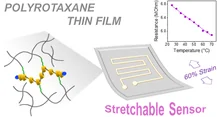

## 1 Introduction

The shift from hard and rigid to soft and flexible electronic systems is revolutionizing interactions between humans and machines, such as in consumer electronics and the Internet of Things [1, 2, 3, 4], soft robotics [5, 6, 7], and interconnected biomedical applications [2, 8, 9]. Indeed, the introduction of flexibility in electronic systems has allowed the development of wearable devices, which can be employed for efficient human motion tracking and immersive experiences in augmented reality [6, 10], as well as for continuous monitoring of physiological signals for disease detection and management [9, 11, 12].

The main challenge for reliable, long‐term operation of flexible and wearable devices on moving substrates, such as skin, is maintaining performance under continuous mechanical deformation during motion [13]. In most cases, a mismatch in mechanical properties between the device and the substrate is the leading cause for various adverse phenomena affecting both the soft moving substrate, which can be irritated or damaged by the poorly adhering device, and the sensing performance, leading to increased noise during data collection and loss of sensing capability [13, 14].

To overcome these limitations, two main strategies have been pursued for optimizing the mechanical reliability and overall performance of flexible electronic systems. One is based on the implementation of a complex design of interconnected architectures and geometry in the device [15, 16, 17] that prevents the platform from failing due to mechanical deformation. The other strategy is based on tuning the composition of polymeric stretchable materials [16, 18, 19], to achieve the desired mechanical properties, before their use as substrates for printing the electrical conductors [20]. The mechanical properties of the polymer substrate are commonly modulated by adjusting the cross‐link density or by introducing reinforcing filler materials [21, 22]. However, devices fabricated from these optimized substrates can still exhibit performance degradation and premature mechanical failure due to the stress concentration caused by nonhomogeneous distribution of cross‐linking junctions [23]. To overcome these limitations, polyrotaxanes (PR) can be used as an alternative polymeric filler to develop highly and reversibly stretchable polymer films. Indeed, PRs are mechanically interlocked molecules, consisting of polymer chains threaded through multiple molecular rings and stoppered at each end [23, 24], resulting in high degrees of conformational freedom and mobility of their components. The incorporation of polyrotaxanes in a polymer network by covalent cross‐linking through their rings provides materials featuring sorts of “molecular pulley,” which demonstrates stress dissipation by the slide‐ring motion [25], and a typical mechanical response characterized by enhanced toughness [26, 27], stretchability, and softness [24, 28]. Furthermore, the addition of PR derivatives into polymeric materials has already demonstrated a promising strategy for developing artificial muscles and sensors [25], flexible bioelectronics [13, 29], and energy storage devices [30, 31] with enhanced stretchability, mechanical robustness, and durability. Previous studies on polyrotaxane‐based materials have primarily focused on bulk elastomers [23, 32], hydrogels [25], or composite systems [33], aimed at improving mechanical properties or enabling actuation functionality. However, the development of freestanding, micrometer‐thick polyrotaxane‐based films designed to provide the mechanical robustness required for printed electronics fabrication while maintaining the flexibility needed to operate under large mechanical deformation remains largely unexplored.

Herein, we introduce a versatile materials design strategy based on polyrotaxanes with controlled degrees of functionalization, incorporated into covalently cross‐linked multi‐arm PEG networks to produce freestanding films with tunable mechanical properties. Unlike previous studies, in which functionalized polyrotaxanes were primarily employed as additives to improve the mechanical properties of conductive polymer materials [13], the present work establishes polyrotaxanes as the mechanically active component of freestanding cross‐linked polymer films. This strategy enables systematic tuning of the substrate mechanical properties through the sliding‐ring architecture while maintaining compatibility with printed electronics fabrication techniques. By independently controlling PR content and cross‐link density, we demonstrate systematic tuning of stiffness, strength, and toughness while maintaining high stretchability. Importantly, we demonstrate that these materials can serve as substrates for temperature sensors that maintain stable operation under large mechanical deformation. This work, therefore, positions polyrotaxane‐based thin films as promising substrates for stretchable printed electronics operating under large deformations.

## 2 Results and Discussion

Polyrotaxanes (PRs) were designed with a polyethylene glycol (PEG) backbone and sliding α‐cyclodextrins (α‐CDs), which act as movable components to impart stretchability. The α‐CDs were functionalized with poly(ethylene glycol) methacrylate (PEGMA) side chains, yielding biocompatible polyrotaxanes suitable for photopolymerization. The PR was synthesized by threading α‐CDs onto a PEG chain (MW 10 000) in a water solution, followed by end‐capping with adamantyl groups, according to the reported procedure [34]. The successful formation of polyrotaxane was confirmed by ^1^H nuclear magnetic resonance (NMR) spectroscopy (Figures S1–S3). In particular, the threading of α‐CDs along the PEG axle was confirmed by the shift in the NMR signals attributed to α‐CD when compared to unthreaded rings. Integration of the proton signals revealed an average of 9 α‐CD units per PR, in reasonable agreement with the value estimated from the molecular weight determined by gel permeation chromatography (GPC) (Table S1).

To enable cross‐linking, the α‐CD units in the polyrotaxane were functionalized with PEGMA side chains (MW 500) at two different degrees of substitution, yielding PR grafted with 4 (PR‐4MA) and 8 (PR‐8MA) PEGMA chains. The PEGMA chains were determined by ^1^H NMR signal integration of the methacrylate protons of the PEGMA (Figures S4–S5) and confirmed by weight estimation from GPC analysis (Table S1). The relatively low polydispersity indices (PDIs) measured for PR (1.28), PR‐4MA (1.36), and PR‐8MA (1.43) indicate that both the threading and subsequent PEGMA grafting steps preserve a relatively narrow molecular weight distribution, consistent with limited variation in the number of threaded α‐CD units and the average degree of functionalization among different polyrotaxanes.

Although the exact distribution of PEGMA side chains cannot be directly determined, the PEGMA substituents are expected to be statistically homogeneously distributed among the α‐CD units within each polyrotaxane because steric repulsion disfavors multiple grafting events on the same ring [35]. In addition, the relatively low PDI obtained by GPC suggests limited variation in the average degree of functionalization among different polyrotaxanes. Polyrotaxane‐based films were fabricated by mixing aqueous solutions of PR‐4MA or PR‐8MA with a 4‐arm acrylate‐terminated poly(ethylene glycol) (4‐arm‐PEG‐A, MW 10 000) (Figure 1).

**FIGURE 1:**
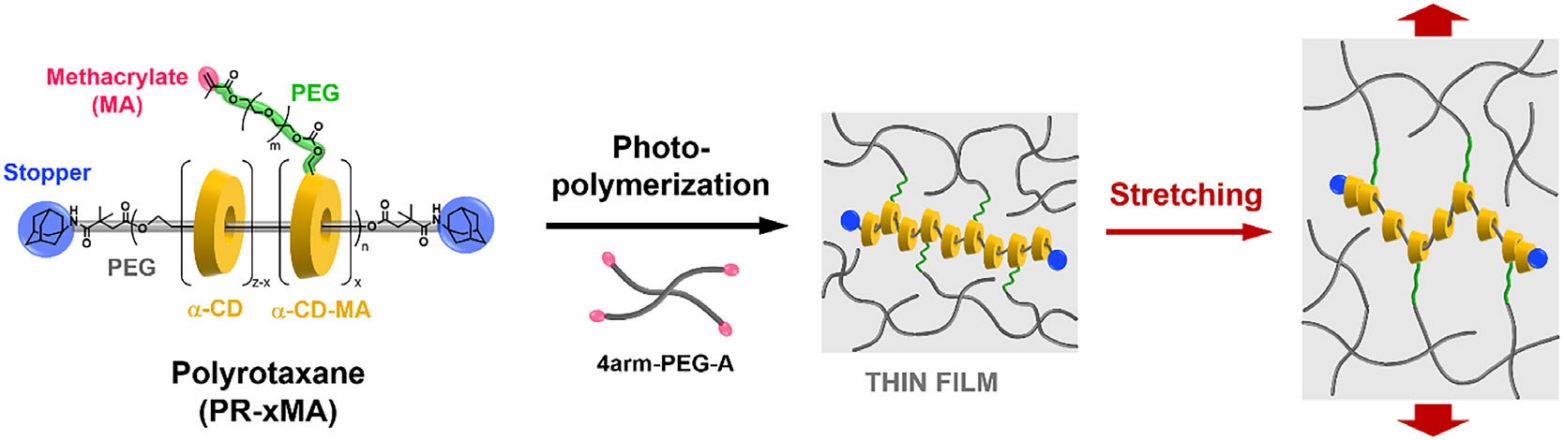
Schematic illustration of polyrotaxanes grafted with poly(ethylene glycol) methacrylate (PEGMA) side chains (*z* indicates the average number of α‐cyclodextrins and *x* the average number of PEGMA units per polyrotaxane), and the subsequent film fabrication by photopolymerization with 4‐arm‐PEG‐acrylate (4‐arm‐PEG‐A). The graphical representation highlights the ring‐sliding motion in the polyrotaxane, which dissipates stress and thereby enhances the stretchability of the polymeric thin film.

The mixtures were cast into Petri dishes and photopolymerized under UV light to produce cross‐linked thin films, followed by air‐drying until reaching low water content (1.0 ± 0.1%). Four films were prepared, corresponding to PR‐4MA and PR‐8MA combined with 4‐arm‐PEG‐A at 1:3 (High) and 1:6 (Low) ratios (Figure 2a). These different feed ratios for polymerization are expressed in terms of the relative number of acrylate units in the grafted PR and 4‐arm‐PEG‐A. The thicknesses of the obtained films range from 8 to 10 µm, as observed by scanning electron microscopy (SEM) (Figure S6). The successful grafting of methacrylate groups from the functionalized polyrotaxanes within the films was confirmed by Fourier transform infrared (FTIR) spectroscopy (Figure S7). The spectra exhibited a characteristic absorption band at 1746 cm^−1^, corresponding to the stretching vibration of the carbonate group from the PEGMA substituents in the PR, confirming the incorporation of PR into the cross‐linked films. In addition, the disappearance of the band at 1640 cm^−1^, assigned to the C=C double bond, indicates successful photopolymerization of the methacrylate groups. Quantitative incorporation of PR‐xMA and 4‐arm‐PEG‐A into all cross‐linked films was demonstrated by ^1^H NMR spectroscopy following alkaline hydrolysis of the acrylate cross‐linking units (Figure S8), with the experimental compositions showing good agreement with the respective monomer feed ratios.

**FIGURE 2:**
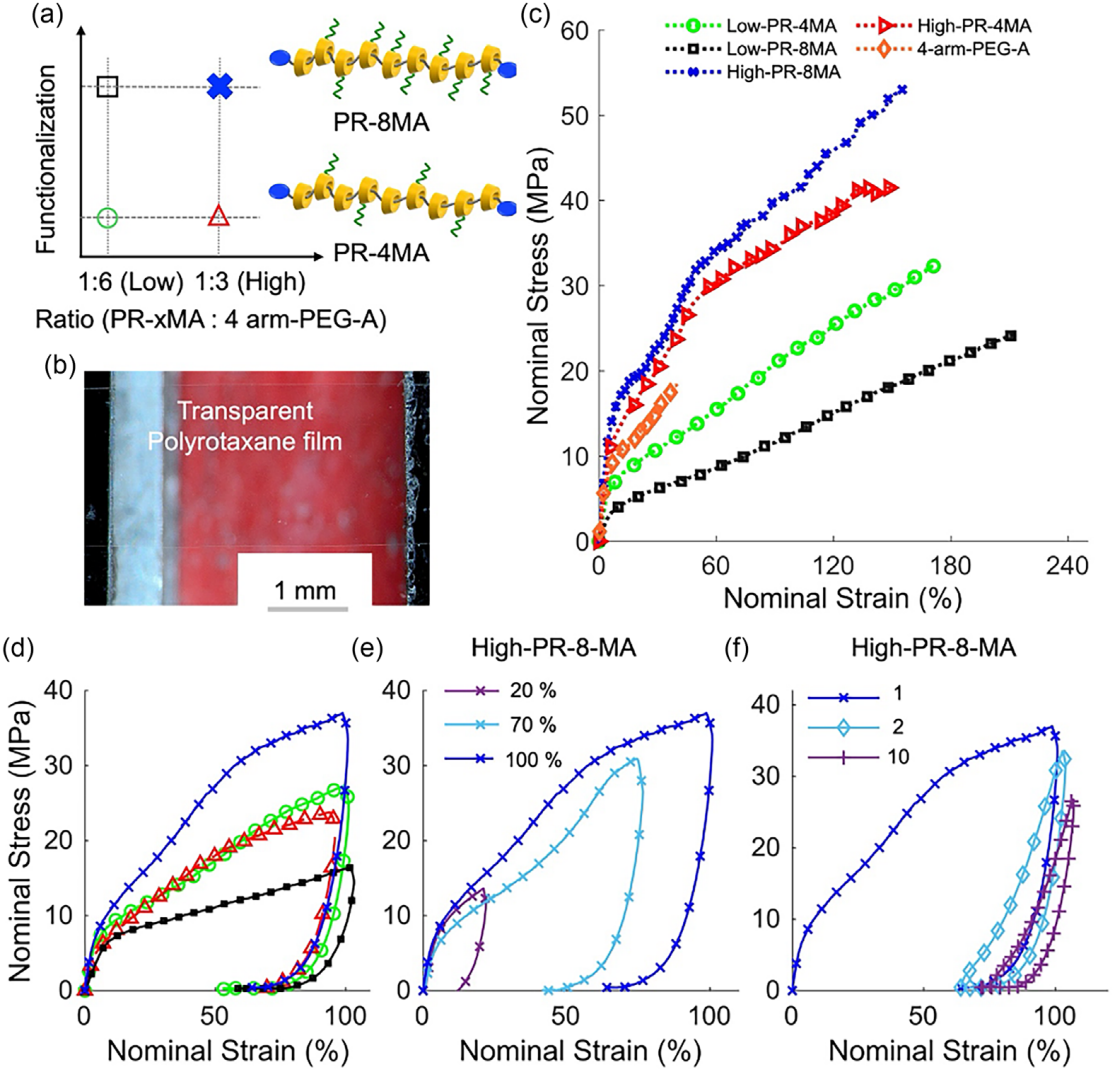
Mechanical response of polyrotaxane thin films. (a) Schematic representation of the two different functionalization degrees of methacrylates (PR‐4MA and PR‐8MA) used for preparing polyrotaxane films having different ratios of acrylate provided by PR‐xMA and 4‐arm‐PEG‐A: Low‐PR‐4MA (1:6), High‐PR‐4MA (1:3), Low‐PR‐8MA (1:6), High‐PR‐8MA (1:3). (b) Image of a freestanding polyrotaxane thin film on the tensile testing platform. (c) Stress–strain response of the films against uniaxial tensile loading up to failure. (d) Stress–strain curves representing the first loading–unloading response of all films at an applied strain amplitude of 100%. (e) Mechanical behavior of High‐PR‐8MA‐film at three different amplitudes of applied strain (20%, 70%, and 100%) corresponding to the first cyclic loading. (f) Response of High‐PR‐8MA film against ten consecutive cyclic loadings.

We then investigated the mechanical properties of the films (Figure 2b) by performing uniaxial tensile tests on a custom‐made platform (Figure S9), yielding the stress–strain curves shown in Figure 2c (all curves are shown in Figure S10). The average values of strength, failure strain, Young's modulus, and toughness differ across the films (Table 1), indicating that their mechanical response is regulated by both the degree of PR functionalization and its relative content in the polymer network, the two key parameters considered during film preparation.

**TABLE 1:** Mechanical properties evaluated by uniaxial tensile tests of the thin films obtained from 4‐arm‐PEG‐A and at different ratios of polyrotaxane to PEG‐A. Standard deviations were obtained from three measurements.

PR film	Failure strain, %	Strength, MPa	Elastic modulus, MPa	Toughness, MJ/m^3^
4‐arm‐PEG‐A	28 ± 10	16 ± 5	226 ± 28	3 ± 2
Low‐PR‐4MA	172 ± 27	33 ± 4	167 ± 16	35 ± 9
High‐PR‐4MA	155 ± 6	41 ± 1	171 ± 44	42 ± 2
Low‐PR‐8MA	201 ± 23	23 ± 5	74 ± 3	27 ± 8
High‐PR‐8MA	162 ± 27	52 ± 1	227 ± 73	54 ± 9

All the investigated PR‐xMA films demonstrated good stretchability, exhibiting comparable failure strains above 150% and significantly higher than the control 4‐arm‐PEG‐A film (28 ± 10%), with the highest value recorded for Low‐PR‐8MA (201 ± 23%). The enhanced deformability of the PR‐based films can be attributed to the sliding motion of the α‐CD rings along the PEG axle, promoting toughness and stretchability when compared to a conventional chemically non‐sliding cross‐linked polymer network [24, 28]. An increase in the PR‐8MA content in the film results in superior load‐bearing capacity and stiffness compared to PR‐4MA (Figure 2c,d). Notably, the Low‐PR‐8MA film exhibits a significantly lower elastic modulus compared to the other films. This behavior can be attributed to the low polyrotaxane content, which reduces the effective cross‐link density and results in a softer network, despite the higher degree of functionalization of the PR units.

Indeed, with respect to Low‐PR‐8MA film, High‐PR‐8MA film displays doubled strength (52 ± 1 MPa) and toughness (54 ± 9 MJ/m^3^), and a 3‐time increase in the Young's modulus (227 ± 73 MPa), equaling the modulus of control 4‐arm‐PEG‐A film, while still maintaining high stretchability owing to the presence of sliding‐ring components. On the contrary, increasing the content of PR‐4MA provides only a slight increase in the strength of the film (41 ± 1 MPa) when compared to Low‐PR‐4MA (33 ± 4 MPa). Finally, it is interesting to compare the mechanical properties of the films obtained from solutions with equal concentrations of PR units, i.e., Low‐PR‐4MA and High‐PR‐8MA, which differ in the number of covalent cross‐linking units (provided by PEGMA‐grafted chains on the PR backbone), which is higher in High‐PR‐8MA. The films exhibit similar failure strains, while the strength, toughness, and Young's modulus of the High‐PR‐8MA film are about 1.5 times those of the Low‐PR‐4MA film, achieved with ~14% increase in the cross‐link density of the film. Our strategy, therefore, provides an effective route for enhancing typically self‐excluding properties, such as strength and toughness, in PR‐based films by systematically tuning network composition and reticulation, which is essential for the development of high‐performance devices. Overall, our results are in good agreement with those reported for PR‐based films [13, 36, 37, 38], and comparable to commercially available polydimethylsiloxane (PDMS), a widely used elastomer for the fabrication of flexible electronic devices. In particular, the optimized PR‐based films exhibit higher strength and toughness, arising from the sliding‐ring mechanism, which promotes efficient stress redistribution and energy dissipation during deformation [26, 27] while maintaining comparable or greater stretchability than PDMS (strain ~100%, strength ~2.3 MPa, toughness 1.1 MJ/m^3^) [39]. With respect to conventional substrates for wearable and stretchable electronics, whose elastic modulus is typically in the range of 1 MPa or below, with the exception of thermoplastic polyurethane (TPU), which can exhibit elastic moduli above 10 MPa [40, 41, 42], our PR‐based films are stiffer yet with comparable stretchability and strength and also offer greater versatility in tuning the mechanical properties through control of several parameters, including polymer backbone composition and molecular weight, ring size, type, and functionalization. Furthermore, other polymeric thin films proposed for wearable and stretchable electronics exhibit Young's moduli in the range from 200 to 2000 MPa, such as semiconducting polymer films exploiting the nanoconfinement effect [19] or other PR‐based films composed of poly(3,4‐ethylenedioxythiophene):polystyrene sulfonate (PEDOT:PSS) and PR‐PEGMA [13].

Regarding the shape of the stress–strain curves, films prepared at low PR content exhibit a monotonic decrease in slope, indicating progressive softening with increasing strain (Figure S11). In contrast, High‐PR‐xMA films display a more complex response. After an initial decrease at small strain, the slope of the curve increases at intermediate strain (~50%), consistent with a strain‐stiffening regime, followed by a subsequent decrease at larger strain prior to failure (Figure S11). This behavior, which has also been reported for polyrotaxanes functionalized with side chains of different lengths [27, 43], suggests that strain stiffening arises from a transition in the dominant deformation mechanism. At low strain, the response is governed by elastic deformation of the network. At higher strain, the sliding α‐CD rings are expected to progressively align along the PEG backbone, leading to increased resistance to further deformation and the onset of strain stiffening [44]. The relatively high stiffness of the films is attributed to the covalently cross‐linked PEG network, whereas the enhanced stretchability and toughness arise from the sliding‐ring mechanism introduced by the incorporated polyrotaxane. The more pronounced effect observed for higher degrees of functionalization (PR‐8MA) can be attributed to the larger number of covalently linked PEGMA chains, which may restrict ring mobility and enhance stiffness under deformation [45]. This interpretation is consistent with previous studies, showing that strain stiffening is strongly governed by ring mobility [44] and is also influenced by the polyrotaxane content within the polymer network [43]. In contrast, Low‐PR‐xMA films do not exhibit strain stiffening, likely due to the lower concentration of polyrotaxane units, which limits cooperative effects such as ring alignment and network reinforcement under strain.

To further elucidate the effect of PR‐xMA on the mechanical properties of the films, cyclic loading–unloading uniaxial tensile tests were performed, and the results are shown in Figure 2d–f and Figure S12. The stress–strain curves for all the films investigated exhibit different responses during loading and unloading, indicating significant dissipative loss in the polymer networks, consistent with dynamic cross‐linking junctions, such as the sliding motion of the polyrotaxane rings [28, 44]. After subjecting the films to consecutive loading–unloading cycles, the stress–strain curves tend to assume a J‐shape (Figure 2f and S13), in agreement with previous observations, supporting the role of ring sliding in stress release [24, 28, 30, 46]. In particular, from the first to the second cycle, some stress softening effects, i.e., a decrease in initial stiffness, can be observed depending on the strain level employed, whereas during the following cycles (second to tenth), no significant differences are observed (Figure S13). From the tests, all the developed PR‐xMA films demonstrated stress recovery greater than 70% after 10 consecutive cycles at a 100% strain amplitude, with no significant differences among the tested conditions. These results therefore indicate that incorporating PR‐xMA through covalent cross‐linking into the films significantly restores the polymer matrix properties during multiple deformations, thanks to the slide‐ring effect, while the absence of significant differences could probably be ascribed to the large amount of PR‐MA employed in the films, hindering the contribution from the grafting degrees of polyrotaxane.

To explore the suitability of PR films as structural materials for printed electronic devices, it is essential to determine the temperature range in which the films retain full functionality. In this regard, we performed differential scanning calorimetry (DSC) analysis of all the films (Figure S14) and observed similar thermal stability up to 220 °C, with decomposition occurring at 260 °C, a typical decomposition temperature for α‐CD/PEG‐based polyrotaxanes [47]. This thermal stability ensures compatibility with common processing conditions used in printed electronics. Based on this result and its superior mechanical performance, the High‐PR‐8MA film was selected as a representative system to evaluate the suitability of PR‐based materials as substrates for stretchable printed electronics. In stretchable electronic devices, the mechanical properties of the substrate play a critical role in device performance under deformation, as strain localization and interfacial stress can lead to failure of the conductive layer. Therefore, substrates that combine high stretchability, mechanical recovery, and thermal stability are essential for stable operation. As a proof of concept, we fabricated temperature sensors with an identical serpentine layout (Figure 3a) using two different conducting materials and fabrication strategies. One sensor is obtained by directly printing a silver (Ag) paste using a motorized nozzle on the High‐PR‐8MA film (Figure 3b), followed by sintering at 120 °C for 30 min. The second sensor is produced by adding a vinyl mask to the High‐PR‐8MA film substrate, followed by drop‐casting an aqueous 0.05% carbon nanotube (CNT) solution, which is then stabilized on a hot plate at 50 °C to form a first CNT layer. The procedure is repeated to form a second layer and then rinsed with DI water. After air‐drying of the substrate, additional drop‐casting is performed until 6 layers form on the High‐PR‐8MA, followed by mask removal from the substrate (Figure 3c). Both the printed and drop‐casted geometrical patterns obtained on the films exhibit excellent matching with the original layout employed (Figure S15). Then, we investigated the effect of temperature change on the resistance of the printed layouts. The Ag‐printed sensor was investigated over the temperature range from 25 °C to 75 °C, showing a linear increase in resistance with increasing temperature (Figure 3d). A positive temperature coefficient of resistance (TCR) of 0.00393 (°C)^−1^ can be obtained, consistent with a metallic conduction mechanism, and similar to the theoretical TCR value for Ag (0.00382 (°C)^−1^) [48]. On the contrary, the CNT‐based sensor obtained by drop‐casting displays a linear decrease in resistance between 30 °C and 70 °C, exhibiting a negative TCR of −0.00302 (°C)^−1^ (Figure 3e), typical of semiconducting materials, and in good agreement with previously reported CNT‐based temperature sensors [49, 50].

**FIGURE 3:**
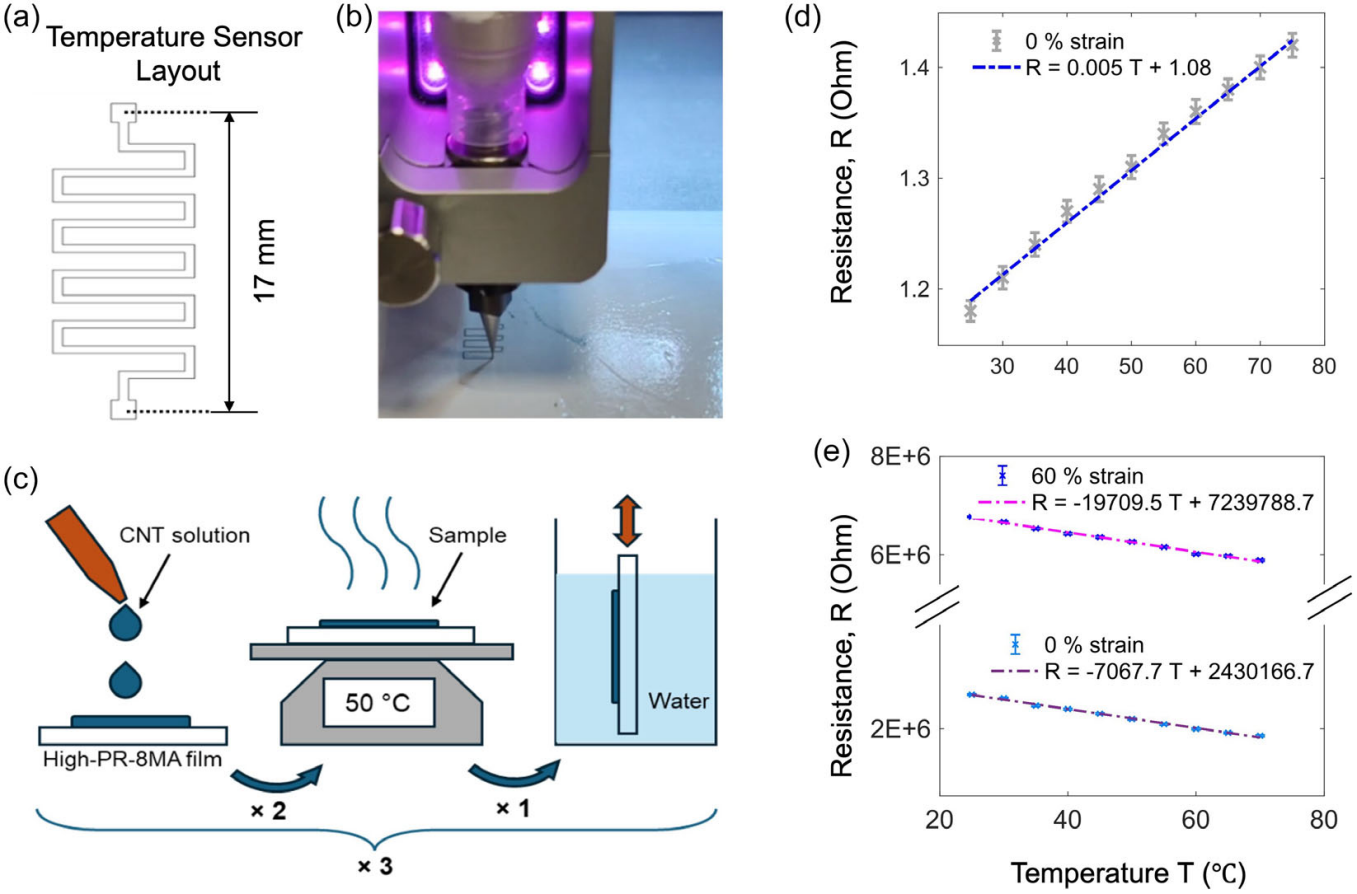
Fabrication and response of temperature sensors on PR‐based films. (a) Layout (with pad‐to‐pad distance) of the meander circuit deposited onto a High‐PR‐8MA film. (b) Image of Ag paste printing on High‐PR‐8MA film through direct ink writing. (c) Schematic representation of CNT‐based sensor fabrication through drop‐casting and rinsing of a masked High‐PR‐8MA film from 0.05% w/w CNT aqueous solution. (d,e) Resistance–temperature curves of temperature sensors fabricated using (d) Ag paste and (e) CNT conductive layers.

When the temperature sensors are stretched in the pad‐to‐pad direction, the Ag‐printed layout rapidly degrades, whereas the CNT‐based platform remains fully functional up to ~60% strain (Figures S16 and S17). This behavior highlights the importance of matching the mechanical properties of the conductive layer with those of the substrate. While metallic conductors, such as Ag, are inherently brittle and prone to failure under deformation, CNT networks can accommodate strain through structural rearrangement, enabling stable operation on a mechanically compliant substrate. Under 60% strain, selected as a representative large deformation at which conventional non‐sliding‐ring materials fail, the resistance of the CNT electrode increases, consistent with a piezoresistive response (Figure 3e).

A linear resistance decrease is preserved in the temperature range 30 °C–70 °C under strain, with only a slight reduction in the TCR (−0.00289 °C^−1^), which remains within a range consistent with literature reports [51].

These results, therefore, demonstrate that the reported polymer films can be employed for the fabrication of stretchable electronic devices, which maintain good performance under significant strain, especially when combined with strain‐tolerant electrode materials.

It is worth noting that the device layout used in this study helps mitigate strain‐induced changes in resistance that can interfere with temperature measurements. In particular, the serpentine geometry was designed to accommodate mechanical deformation while minimizing its influence on the electrical resistance. To further improve the decoupling of temperature and strain, a more sophisticated device layout needs to be adopted, for example, combining linear resistors oriented along two orthogonal directions with a serpentine resistor.

The PR films developed here are promising as substrates for wearable electronics. Indeed, their high stretchability (>150%) may enable them to withstand the large deformations associated with joint movement, such as local strain of approximately 30%–50% during knee or elbow flexion, without breaking [52]. Furthermore, their relatively high stiffness can reduce the device's susceptibility to unwanted deformations (such as those caused by small external mechanical loads), which may alter the electrical resistance and complicate sensor readings, thereby contributing to a more stable electrical baseline during operation.

## 3 Conclusion

In summary, this study demonstrates a versatile materials design strategy for tuning the mechanical properties of cross‐linked polymer films by incorporating polyrotaxanes into covalently cross‐linked multi‐arm PEG networks. Unlike conventional polyrotaxane‐based systems, in which polyrotaxanes are primarily employed as functional additives within polymer matrices, the present work establishes them as the mechanically active structural component of freestanding, micrometer‐thick cross‐linked substrates for stretchable electronics. Consequently, the enhanced stretchability, strength, and toughness arise directly from the sliding‐ring architecture rather than from conductive composites or multilayer elastomeric architectures. By varying the polyrotaxane content and the degree of functionalization, the mechanical response of the films can be systematically tuned, enabling the combination of high stretchability with enhanced strength and toughness, while still providing ample opportunities for further tailoring through control of the polymer backbone composition and molecular weight, ring size, ring type, and degree of functionalization.

The resulting films were successfully employed as substrates for stretchable temperature sensors, demonstrating stable operation under strains up to 60% when combined with CNT‐based conductive materials. Overall, the polyrotaxane‐based thin films combine stiffness and stretchability, bridging the gap between conventional flexible plastics, such as polyethylene terephthalate (PET) and polyimide (PI), and soft elastomers (PDMS, TPU). These results establish them as mechanically robust and processable platforms for stretchable printed electronics, where the substrate's mechanical properties play a critical role in maintaining device functionality under deformation.

## 4 Experimental Section

### 4.1 Reagents and Materials

All reagents were purchased from commercial suppliers and used without further purification. Four‐arm acrylate‐terminated poly(ethylene glycol) (4‐arm‐PEG‐A, MW 10 000) was purchased from JenKem Technology USA (Plano, TX). All other reagents were purchased from Sigma‐Aldrich Co. (St. Louis, MO).

### 4.2 Instrumentation

NMR experiments were performed using a Bruker 400 MHz Avance III HD, operating at 400.13 MHz for ^1^H experiments, equipped with a BBI‐z grad 5 mm probehead. The NMR spectra were processed using Topspin 3.5 software, and the chemical shift scale was calibrated using the residual solvent signal. FTIR spectra were obtained with an FTIR Nicolet Nexus 670 spectrometer. GPC measurements were performed using a Shimadzu Shim‐Pack GPC‐80MD column equipped with an RID‐20A refractive index detector. Analyses were carried out at 25 °C with dimethylformamide (DMF) as the eluent, at a flow rate of 1.0 mL/min, and using narrow‐dispersity linear polyethylene glycol (PEG) standards (American Polymer Standards Corporation) for calibration. The film polymerization was performed with a UV lamp from Analitik Jena. SEM images were taken on a JEOL IT300 microscope. DSC analysis was performed with a DSC 25 instrument (TA Instruments, New Castle, DE). Tensile tests were performed with a custom‐made platform consisting of a piezoelectric motor (actuator) and a calibrated spring (load sensor), which were glued to a silicon slice, each supporting one end of the film sample to be tested. The testing stage used herein is an adapted version of the platform earlier reported by us [53].

## 5 Methods

### 5.1 Preparation of Polyrotaxane‐Based Films

Polyrotaxane‐based films were fabricated in glass Petri dishes (diameter: 29 mm) by UV photopolymerization. Briefly, 37.5 mg of 4‐arm‐PEG‐acrylate and 4.5 mg of 2‐hydroxy‐4′‐(2‐hydroxyethoxy)‐2‐methylpropiophenone (photoinitiator) were dissolved in 1.5 mL of deionized water. To this solution, different amounts of functionalized PR were added, depending on the different PR‐to‐4‐arm‐PEG‐acrylate ratio (Table 2). The ratios refer to the relative number of acrylate units in PR‐xMA and 4‐arm‐PEG‐acrylate. The mixtures were stirred until complete solubilization, degassed under Ar, and poured into Petri dishes. Photopolymerization was carried out under 365 nm UV light for 30 min. The resulting films were thoroughly washed with deionized water to remove unreacted species and dried at 35 °C for 2 days.

**TABLE 2:** Amounts of functionalized PR used for film preparation at different PR‐xMA:4‐arm‐PEG‐acrylate ratios, expressed in terms of acrylate units. The corresponding concentrations of PR‐xMA in the solutions are reported in brackets.

	PR‐xMA:4‐arm‐PEG‐A	
	1:6	1:3
PR‐4MA	13.1 mg (0.42 mM)	26.4 mg (0.84 mM)
PR‐8MA	7.2 mg (0.21 mM)	14.4 mg (0.42 mM)

### 5.2 Preparation of 4‐arm‐PEG‐A‐Based Films

The control 4‐arm‐PEG‐A film was prepared with the same total amount of acrylate units as the films fabricated at the high PR‐to‐4‐arm‐PEG‐A ratio (1:3). Briefly, 4‐arm‐PEG‐A (50.1 mg) and 2‐hydroxy‐4′‐(2‐hydroxyethoxy)‐2‐methylpropiophenone (4.5 mg) were dissolved in DI water (1.5 mL), and the solution was degassed before casting into glass Petri dishes (diameter: 29 mm). The films were photopolymerized under UV irradiation at 365 nm for 30 min. After polymerization, the films were rinsed with DI water and air‐dried at 35 °C for 2 days.

### 5.3 Water Content Estimation

Water content was estimated according to a reported procedure [54] using the following equation

$$\mathrm{WC}=\frac{m_{s}-m_{d}}{m_{s}}\cdot 100$$

where WC is the water content, *m*_d_ is the mass of the dried polymer powder employed for film formation, and *m*_s_ is the mass of the polymer films employed for characterizations following photopolymerization reaction, washing with DI water to remove unreacted species and air‐drying at 35 °C for 2 days.

### 5.4 Determination of PR‐xMA:4‐arm‐PEG‐A Ratio by ^1^H‐NMR Spectroscopy

The PR‐xMA:4‐arm‐PEG‐A ratio in the cross‐linked films was determined by ^1^H NMR spectroscopy following alkaline hydrolysis of the films, according to a reported method with minor modifications [55]. Briefly, cross‐linked polymer films (~5 mg) were immersed in 700 μL of D_2_O in the presence of 2.0 M NaOD, and the mixture was stirred for 6 h at 60 °C to ensure complete dissolution of the film. After cooling to room temperature, the excess alkali was neutralized by the addition of concentrated deuterium chloride, following transfer of the solution to an NMR tube, and recording of the ^1^H NMR spectrum.

### 5.5 Sensors Fabrication

Two temperature sensors with identical meander design were fabricated on High‐PR‐8MA as substrate film, featuring 450 μm line width, 1.05 mm pitch, 1.125 × 1.125 mm^2^ square pads, a pad‐to‐pad distance of 17 mm, and a total length of 71.625 mm (Figure S15). The first sensor was printed using a Voltera Nova system (Voltera Inc., Canada) equipped with a 225‐μm nozzle, employing Voltera Conductor 3 silver ink (Voltera Inc., Canada). After the ink deposition, a sintering at 120 °C for 30 min was carried out. The second sensor was produced by a masked drop‐casting technique. Vinyl masks were cut with a ScanNCut SDX225 (Brother, USA) and applied to the polymer substrate. The mask‐protected film was drop‐casted using a 0.05 wt% CNT aqueous dispersion containing 0.5 wt% sodium dodecyl sulfate as a surfactant, through six cycles, washing the samples with deionized water every two cycles, according to the reported procedure [56]. Each layer was stabilized on a hot plate at 50 °C. After the final rinsing and drying, the mask was gently removed from the film. The fabricated sensors are shown in Figure S15.

### 5.6 Sensors Investigation

The printed and drop‐casted sensors were placed in a Linkam LTS420 temperature‐controlled stage (Linkam Scientific Instruments, UK) and fixed at the ends using Kapton tape. A 250‐μm‐thick Kapton sheet was placed under the sample to ensure electrical insulation from the probe plate. Two probe tips were positioned at the center of the contact pads, and *I*–*V* curves were recorded using an Agilent E5263A 2‐channel IV Analyzer (Keysight Technologies, USA), sweeping from 0 to 1 V through 101 points. Measurements were performed at 5 °C intervals between 25 °C and 75 °C, maintaining the device at constant temperature for 5 min to achieve thermal equilibrium. Resistance was calculated as the mean value in the 0.3–1 V range. The TCR of the temperature sensors was calculated as $\alpha =\frac{R_{T\text{2}}-R_{T\text{1}}}{R_{T\text{1}}(T_{\text{2}}-T_{\text{1}})}$, where α is the TCR value, *R*_*T*1_ is the electrical resistance of the sensor at the lower relevant working temperature, *R*_*T*2_ is the electrical resistance of the sensor at the higher relevant working temperature, and *T*_1_ and *T*_2_ are the lower and higher relevant working temperature, respectively.

## Funding

This work was supported by the European Union‐Next Generation EU (CUP E53D23005260001).

## Conflicts of Interest

The authors declare no conflicts of interest.

## Supporting information

The Supporting Information is available free of charge. Synthesis of the polyrotaxanes, characterization of the polyrotaxanes by NMR spectroscopy and GPC, characterization of the films by FTIR spectroscopy, tensile tests, thermal analysis, and testing of temperature sensors.

## Data Availability

The data that support the findings of this study are available in the Supporting Information of this article.
