# Supplementary material for: Highly Stretchable Polyrotaxane Thin‐Film Substrates for Printed Electronics
Source: Small Sci. 2026 Oct 16;6(10):e70412. doi: 10.1002/smsc.70412 (PMC13673407; doi:10.1002/smsc.70412)
Supplement: Supplementary file 1 [file SMSC-6-e70412-s001.pdf]

Supporting Information

Highly Stretchable Polyrotaxane Thin-Film Substrates for Printed Electronics

Vivek Manish,[†,‡] Roberto Baretta,[§,‡] Giada Bombardelli,[ǁ,Δ] Guglielmo Trentini,[ǁ,Δ] Chiara Morano,[⊥] Andrea Gaiardo,[ǁ] Luisa Petti,[Δ] Alvise Bagolini,[ǁ] Leonardo Pagnotta,[⊥] Marco Frasconi,[§] Maria F. Pantano[*,†]

[†]Department of Civil, Environmental and Mechanical Engineering, University of Trento, via Mesiano 77, 38123 Trento, Italy

[§]Department of Chemical Sciences, University of Padova, via Marzolo 1, 35131 Padova, Italy

[ǁ]Center for Sensors and Devices, Bruno Kessler Foundation, via Sommarive 18, 38123 Trento, Italy

[Δ]Faculty of Engineering, Free University of Bolzano-Bozen, via Buozzi 1, 39100 Bolzano, Italy

[⊥]Department of Mechanical, Energy and Management Engineering, University of Calabria, via P. Bucci 44C, 87036 Rende (CS), Italy

[‡] These authors contributed equally to this work.

Table of Contents

1. Methods…………………………………………………………………………...S2

2. Film Characterizations………………………………………………………….S11

3. Temperature Sensor Fabrication………..……………………………………S22

4. References……………………………………………………………………..S25

1. Methods

1.1 Synthesis of Polyrotaxanes

The following synthesis was performed according to a reported procedure with minor modifications. [1]

1.1.1 PEG(COOH)$_2$

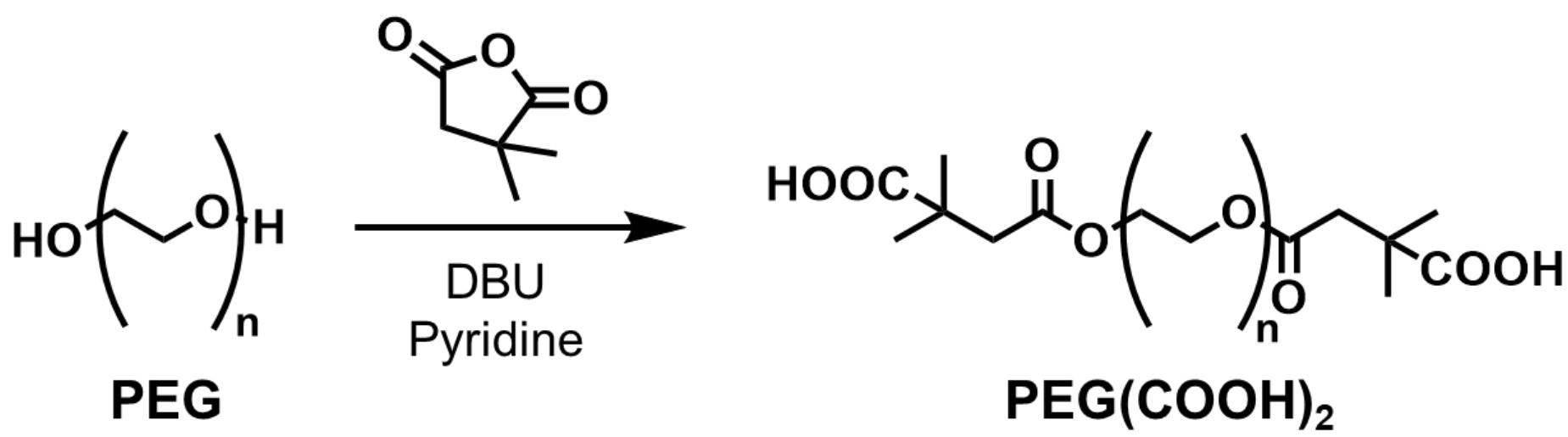

Scheme S1. Synthesis of PEG(COOH)$_2$.

Under argon (Ar) atmosphere, polyethylene glycol (PEG, MW 10000, 7.33 g, 0.73 mmol, 1 eq) was dissolved in 37 ml of anhydrous pyridine, followed by the addition of 1,8-diazabicyclo[5.4.0]undec-7-ene (DBU, 183 μl, 1.20 mmol, 1.6 eq) and 2,2-dimethylsuccinic anhydride (98%, 5.0 g, 38.24 mmol, 52 eq). The solution was kept under magnetic stirring at 60 °C overnight under an Ar atmosphere and then transferred dropwise into 150 ml of DI water. After adjusting the pH to 2 by the addition of concentrated hydrochloric acid (37%), the product was extracted with dichloromethane (DCM). The organic phase was dried with anhydrous sodium sulfate prior to concentration in vacuo, providing PEG(COOH)$_2$ (7.00 g) as a white solid in 93% yield. ^{1}H NMR (400 MHz, DMSO-d_6, 298 K) δ: 12.15 (s, 2H); 4.17-3.38 (m, 940H); 1.17 (12H).

1.1.2 PEGMA-CDI

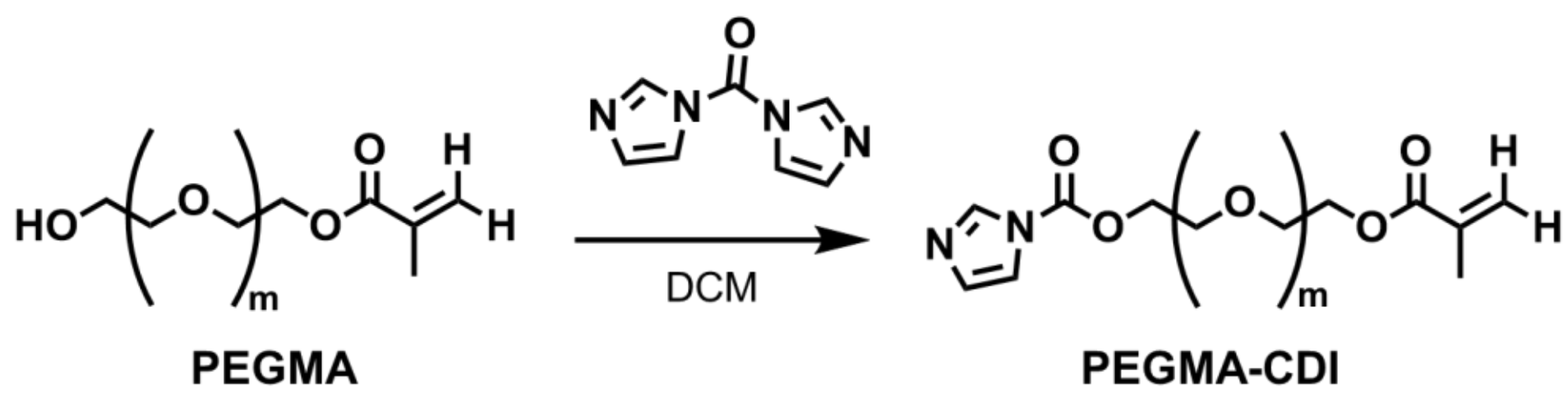

Scheme S2. Synthesis of PEGMA-CDI.

Under an Ar atmosphere, poly(ethylene glycol) methacrylate (PEGMA, MW 500, 2.00 g, 4.00 mmol, 1 eq) was added to 30 ml of anhydrous DCM, and the resulting solution was added to a 50 ml solution of anhydrous DCM containing 1,1'-carbonyldiimidazole (CDI, 0.720 g, 4.35 mmol, 1.09 eq). The resulting mixture was kept under magnetic stirring for 24 h at room temperature under an Ar atmosphere. The mixture was washed with saturated sodium chloride solution, and the collected organic phase was dried with anhydrous sodium sulfate prior to concentration in vacuo, providing an orange viscous liquid PEGMA-CDI (2.15 g) in 90% yield. ^{1}H NMR (400 MHz, $CDCl_3$, 298 K) δ: 8.15 (s, 1H); 7.44 (s, 2H); 7.06 (s, 1H); 6.12 (s, 1H); 5.67 (s, 1H); 4.55 (t, 2H, J = 5 Hz) 4.29 (t, 2H, J = 5 Hz); 3.91-3.56 (m, 40H); 1.94 (s, 3H).

1.1.3 Polyrotaxane

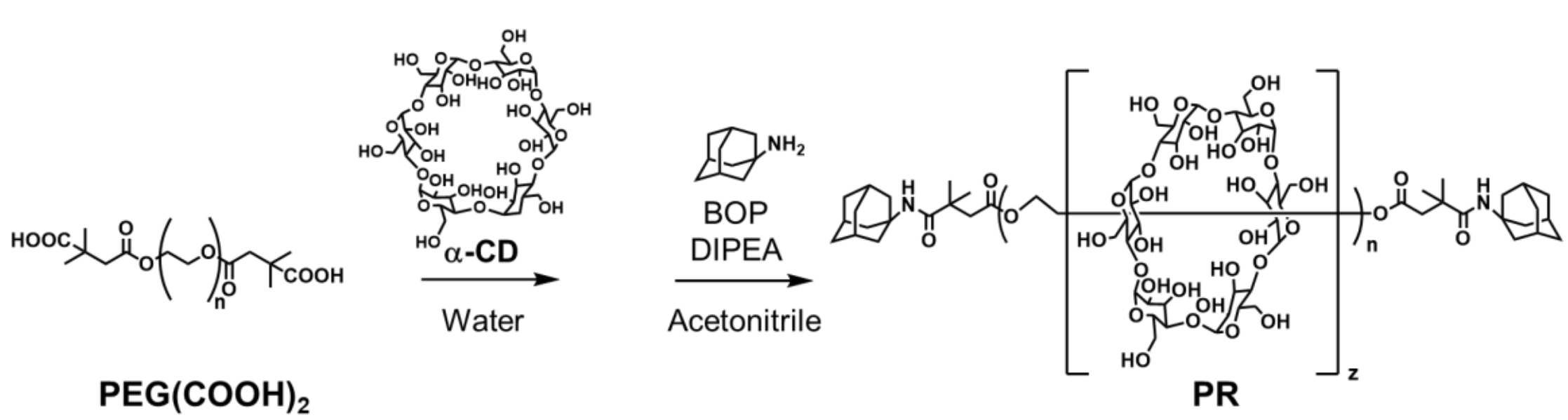

Scheme S3. Synthesis of polyrotaxane (PR).

$PEG(COOH)_2$ (1.50 g, 0.15 mmol, 1 eq) was dissolved in 10 ml DI water, and the solution was combined with 44 ml DI water solution containing α–cyclodextrin (α-CD, 6.00 g, 6.17 mmol, 41 eq). The resulting mixture was stirred for 1 h at room temperature and then stored for two days at 4 °C, leading to the formation of white precipitate. The resulting mixture was then freeze-dried to obtain a white powder. The collected white powder was added to 60 ml of anhydrous acetonitrile under Ar atmosphere and combined with 15 ml solution of anhydrous acetonitrile comprising 1-adamantylamine (97%, 200 mg, 1.28 mmol, 8.6 eq), benzotriazole-1-yl-oxy-tris-(dimethylamino)-phosphonium hexafluorophosphate (BOP, 585 mg, 1.28 mmol, 8.6 eq) and N-ethyldiisopropylamine (DIPEA, 98%, 228 μl, 1.31 mmol, 8.7 eq). The resulting suspension was stirred at room temperature for 48 h under an Ar atmosphere, prior to centrifugation (6000 rpm, 5 min) to remove the supernatant. The white solid collected after centrifugation was washed twice with acetonitrile and dried under vacuum, prior to solubilization in 50 ml of dimethyl sulfoxide (DMSO). The 50 ml DMSO solution was then added dropwise to 500 ml DI water under vigorous magnetic stirring and the resulting aqueous solution was purified by dialysis tubing for 1 week. After dialysis, the remaining solution was freeze-dried to obtain the polyrotaxane composed of α-cyclodextrins threaded through a PEG chain (2.42 g) as a white solid in 86% yield. ^{1}H NMR (400 MHz, DMSO-d_6, 298 K) δ: 5.60 (d, 54H; J = 6.2 Hz); 5.45 (s, 56H); 4.79 (s, 57H); 4.42 (s, 56H); 3.86-3.62 (m, 180 H); 3.62-3.47 (m, 990H); 3.46-3.39 (m, 80 H); 1.98 (s; 6H); 1.95-1.86 (m, 10H); 1.60 (s, 9H); 1.23 (s, 5 H); 1.18-1.09 (m, 12H).

1.1.4 Functionalized Polyrotaxanes

The general procedure for the functionalization of PR polyrotaxane with PEGMA-CDI sidechains involves the solubilization of 200 mg of PR (10.37 μmol, 1 eq) in 4 ml of anhydrous DMSO under

an Ar atmosphere. Different equivalents of PEGMA-CDI (as detailed below) and 4-(dimethylamino)pyridine (DMAP, 0.05 eq relative to PEGMA-CDI) were then added to the solution. The reaction mixture was stirred at 35 °C under an Ar atmosphere for 3 days. After the reaction, the mixture was dropwise added to 40 ml of deionized water. The resulting aqueous solution was purified by dialysis for 1 week, after which it was freeze-dried to yield the polyrotaxane grafted with different PEGMA chains (PR-xMA).

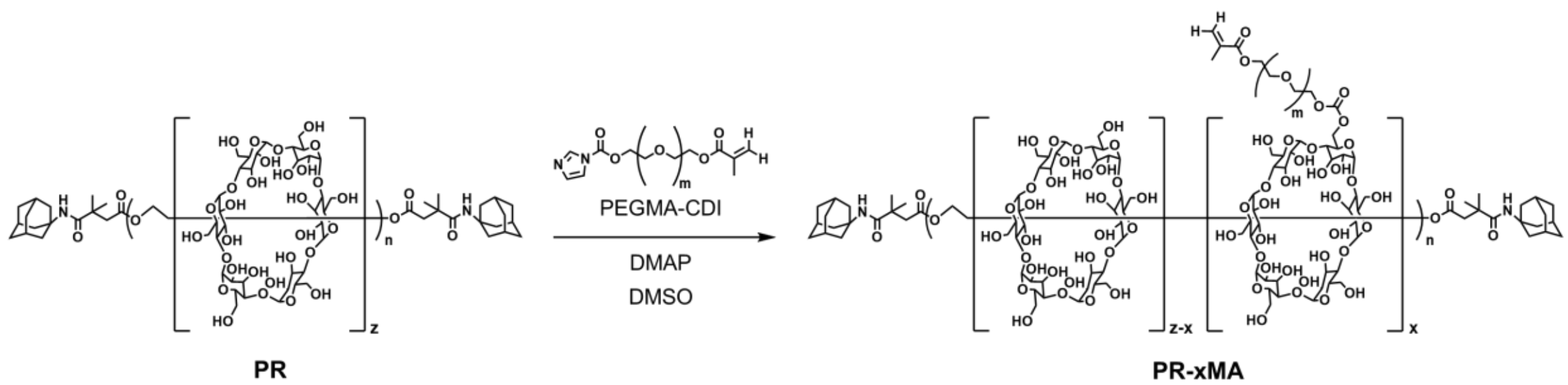

Scheme S4. Synthesis of polyrotaxane functionalized with a different number of PR-PEGMA sidechains (PR-xMA).

1.1.4.1 Low-Density Grafting Polyrotaxane (PR-4MA)

To obtain CD–PR with a low density of grafted PEGMA chains (PR-4MA), 277 mg of PEGMA-CDI (0.47 mmol, 5 equivalents to CD) and 2.9 mg of DMAP (23.74 μmol) were added to the 4 ml anhydrous DMSO solution containing 200 mg of CD–PR (10.37 μmol, 1 eq). After dialysis purification and freeze-drying, 210 mg of white solid was obtained (95% yield). ^{1}H NMR (400 MHz, DMSO-d_6, 298 K) δ: 6.03 (s, 4H); 5.69 (s, 4H); 5.66-4.29 (m); 4.29-3.33 (m, 1040 H); 1.98 (s; 6H); 1.95-1.82 (m, 22H); 1.60 (s, 6H); 1.29-1.02 (m, 22H).

1.1.4.2 High-Density Grafting Polyrotaxane (PR-8MA)

For the high-density grafting (PR-8MA), 555 mg of PEGMA-CDI (0.93 mmol, 10 equivalents to CD) and 5.7 mg of DMAP (46.66 μmol) were added to the 4 ml anhydrous DMSO solution containing 200 mg of CD–PR (10.37 μmol, 1 eq). After dialysis purification and freeze-drying, 230 mg of white solid was obtained (94% yield). ^{1}H NMR (400 MHz, DMSO-d_6, 298 K) δ: 6.03 (s, 8H); 5.69 (s, 11H); 5.66-4.26 (m); 4.26-3.39 (m, 1188H); 1.98 (s; 6H); 1.95-1.82 (m, 38H); 1.60 (s, 6H); 1.31-1.02 (m, 24H).

1.2 NMR Spectroscopic Analysis

1.2.1 PEG(COOH)$_2$

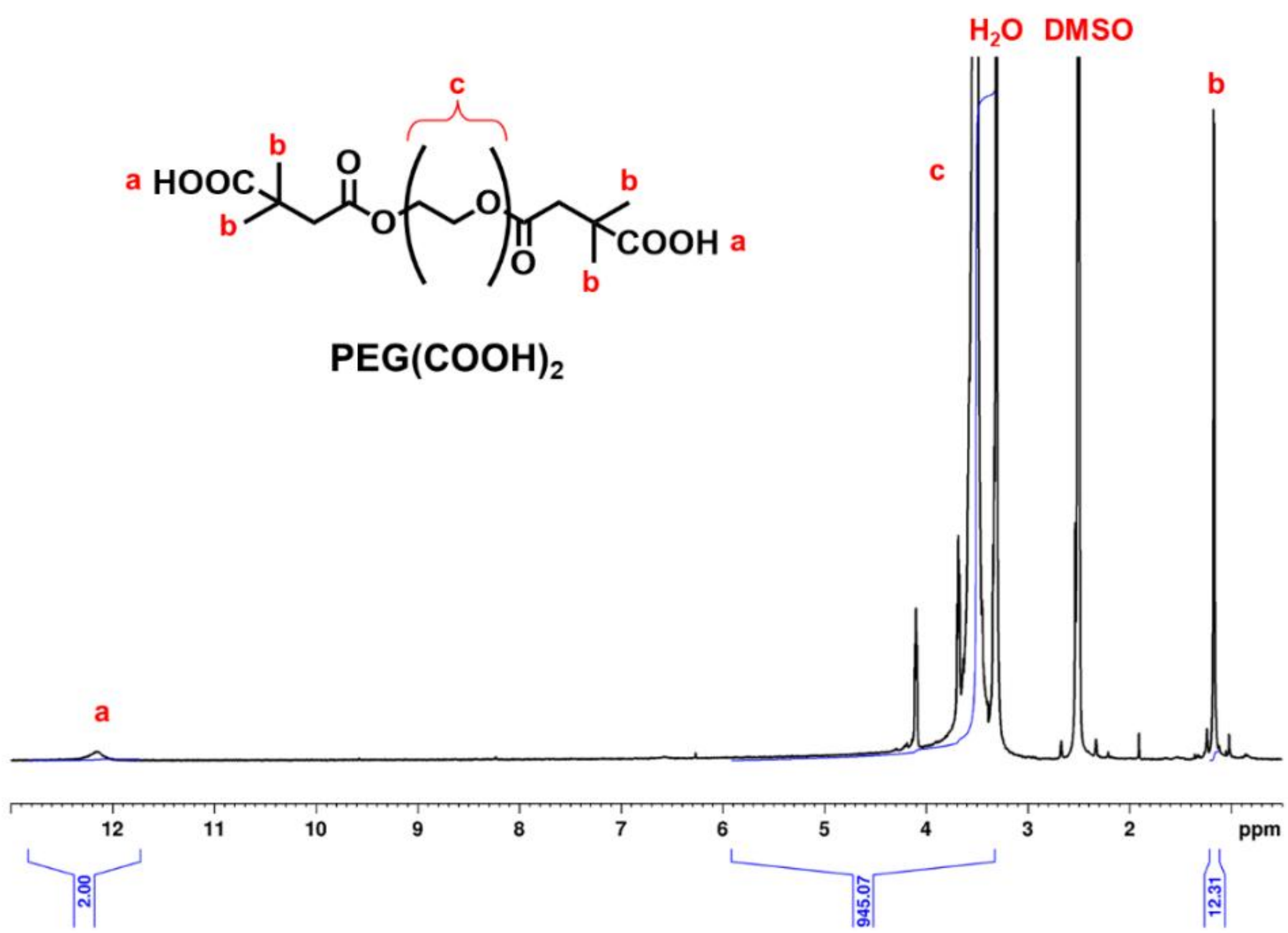

Figure S1. ^{1}H NMR spectrum (400 MHz, DMSO-d_6, 298 K) of PEG(COOH)$_2$.

1.2.2 PEGMA-CDI

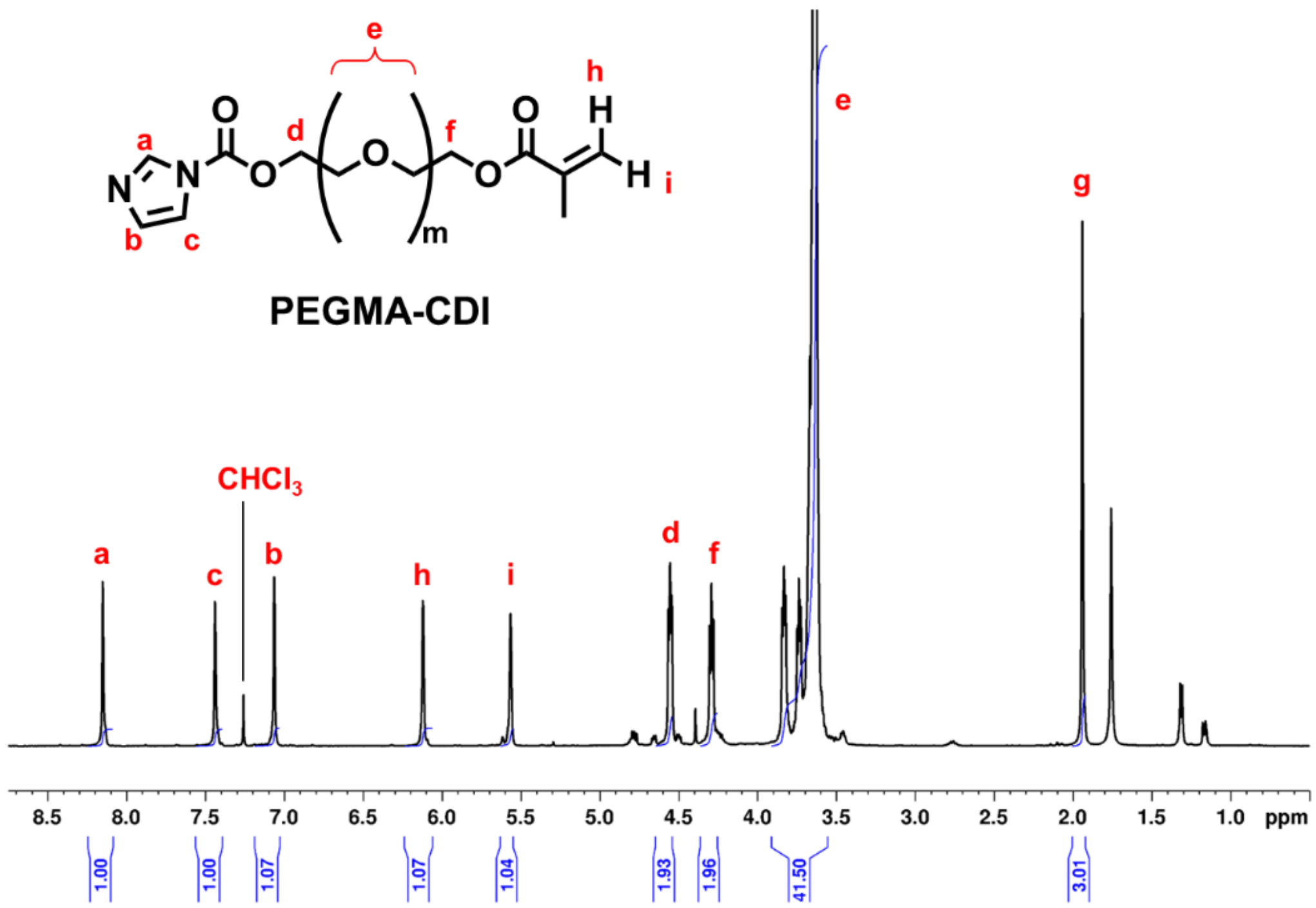

Figure S2. ^{1}H NMR spectrum (400 MHz, $CDCl_3$, 298 K) of PEGMA-CDI.

1.2.3 Polyrotaxane

The formation of polyrotaxane, composed of α-cyclodextrins (α-CDs) threaded onto a PEG chain, was confirmed by ^{1}H NMR spectroscopy (Figure S3), in agreement with previous reports.[1,2] The signals corresponding to the $PEG(COOH)_2$ dumbbell capped with adamantane groups were observed. The threading of α-CDs along the PEG axle was evidenced by characteristic shifts in the α-CD proton resonances compared to those of unthreaded α-CDs.[3]

The average number of α-CDs threaded per PEG chain was estimated from the integral of the proton signal **e**, divided by 6 (the number of equivalent **e** protons per α-CD), resulting approximately in 9 α-CD units per PR.

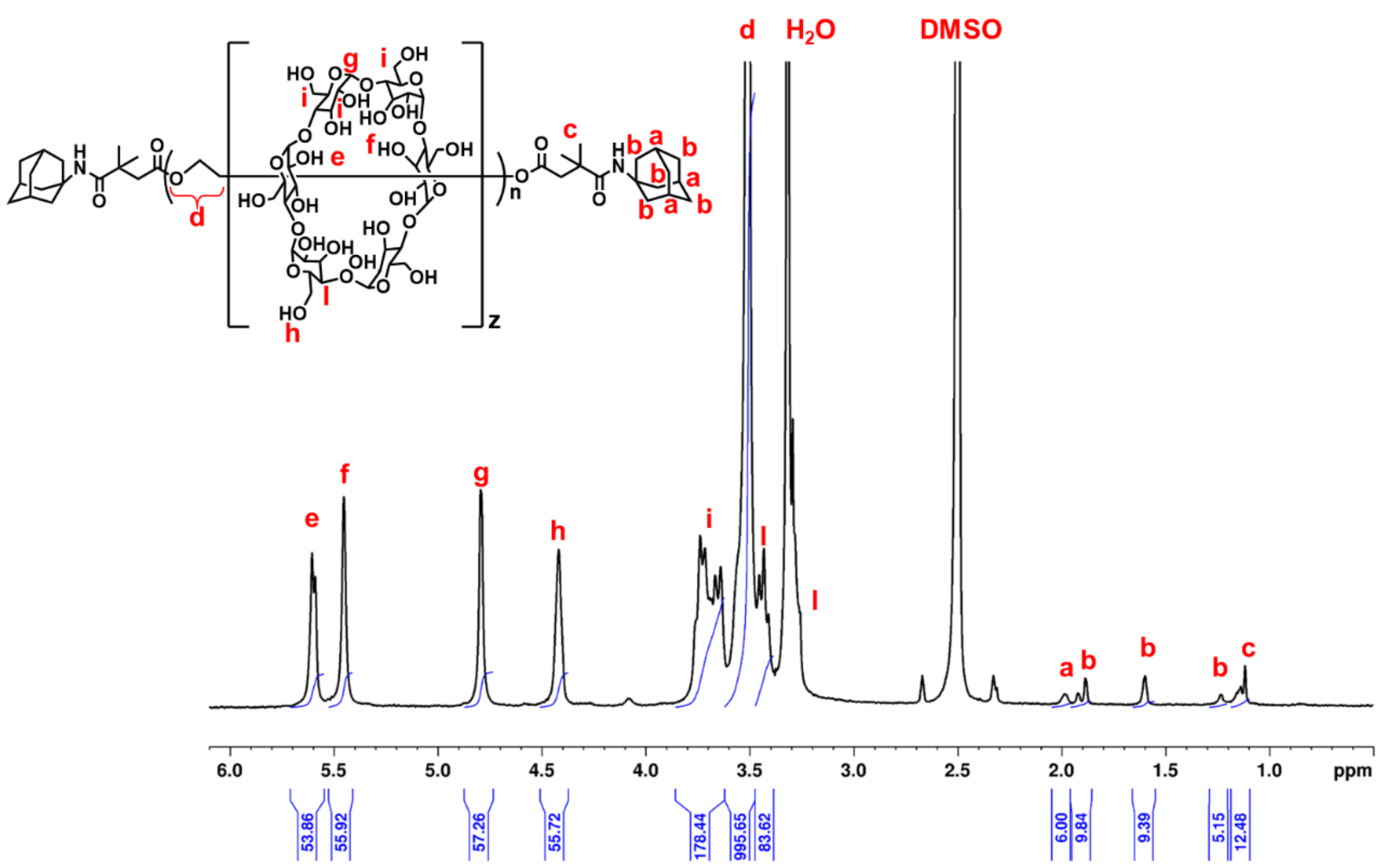

Figure S3. ^{1}H NMR spectrum (400 MHz, DMSO-d_6, 298 K) of polyrotaxane.

1.2.4 Functionalized Polyrotaxanes

The functionalization of PR with different degrees of PEGMA was confirmed by ^{1}H NMR spectroscopy (Figures S4 and S5). The spectra displayed characteristic signals of the methacrylate units (f, e). In addition, the resonances assigned to α-CD protons (h) exhibited noticeable changes in shape and chemical shift, consistent with the covalent functionalization of the threaded cyclodextrins with PEGMA side chains.

The average number of PEGMA groups per polyrotaxane was determined by integrating the alkenyl proton signal of methacrylate (f), using the adamantane –CH protons (a) as an internal reference (set to 6). This analysis indicated that the PR samples contained, on average, 4 (PR-4MA, Figure S4) and 8 (PR-8MA, Figure S5) methacrylate substituents per PR.

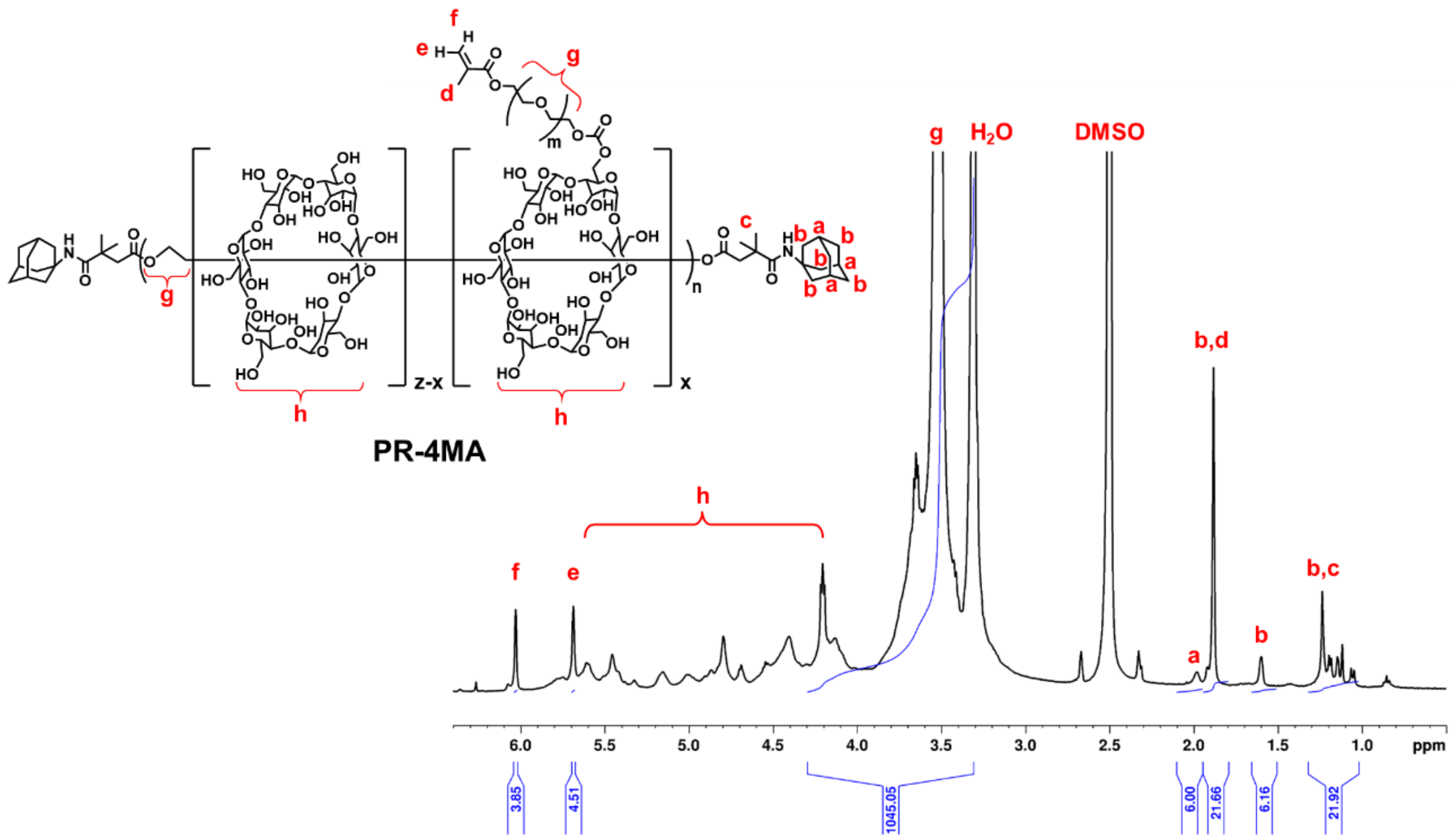

Figure S4. ^{1}H NMR spectrum (400 MHz, DMSO-d_6, 298 K) of PR-4MA.

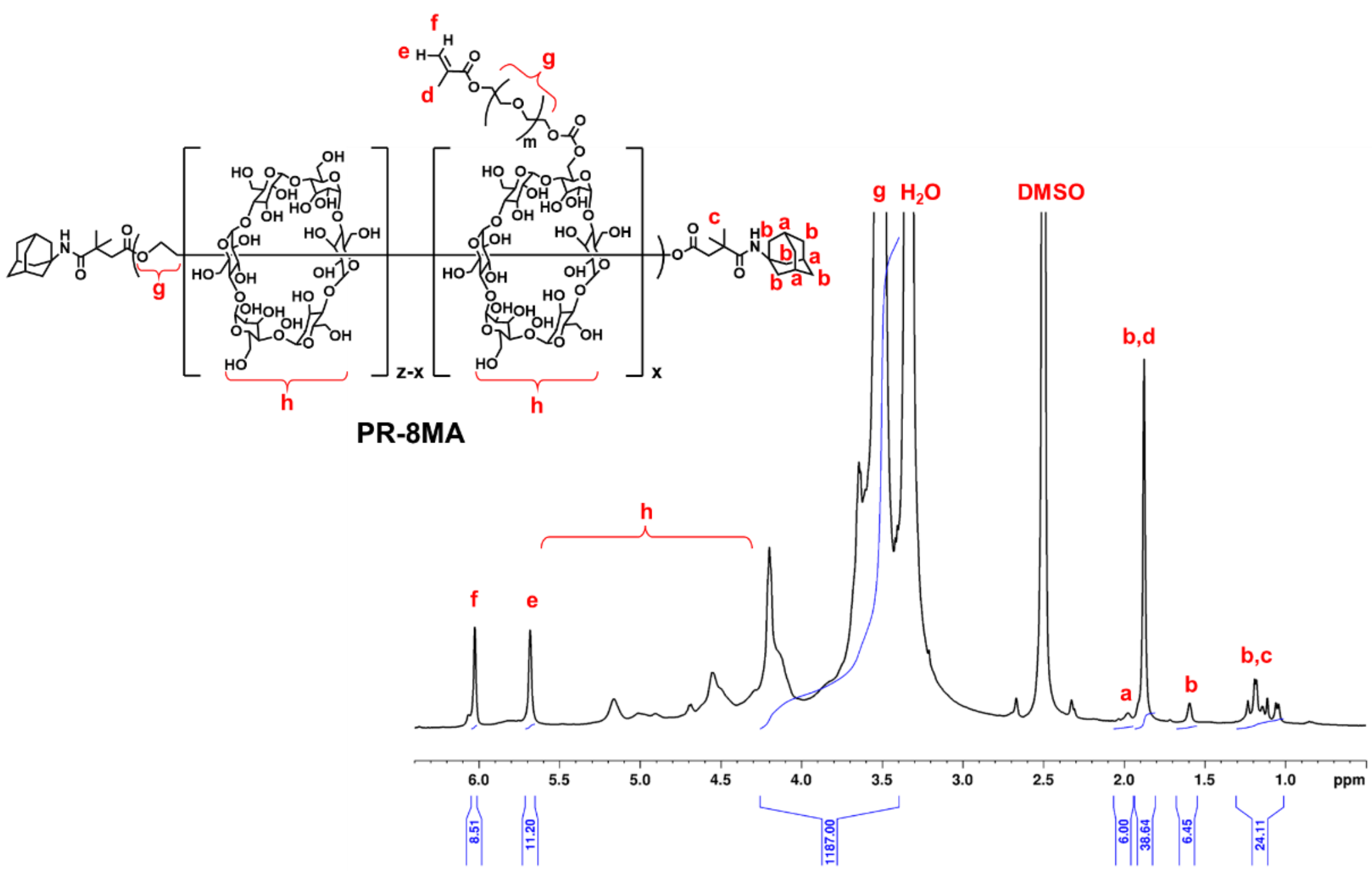

Figure S5. ^{1}H NMR spectrum (400 MHz, DMSO-d_6, 298 K) of PR-8MA.

1.2.5 Gel Permeation Chromatography (GPC) Analysis

Gel permeation chromatography (GPC) of $PEG(COOH)_2$, PR, PR-4MA, and PR-8MA was performed to evaluate their molecular weight distributions, and the results are reported in Table S1.

Table S1. Number average molecular weight (M_n), weight average molecular weight (M_w), and polydispersity index (PDI) of polymer chains.

Sample	M_n (Da)	M_w (Da)	PDI
PEG	10352	11380	1.10
$PEG(COOH)_2$	10490	11850	1.13
PR	17430	22310	1.28
PR–4MA	19310	26260	1.36
PR–8MA	21515	30760	1.43

Compared with $PEG(COOH)_2$, PR displayed an increased molecular weight while maintaining a relatively low polydispersity index (PDI), confirming successful formation of the polyrotaxane. The narrow molecular weight distribution is consistent with limited variation in the number of threaded α-CD units among different polyrotaxane molecules. The average number of threaded α-CD units was estimated from the difference between the number-average molecular weights of PR and the $PEG(COOH)_2$ axle, divided by the molecular weight of a single α-CD (972.84 Da). This analysis yielded an average of approximately seven α-CD units per PR, in reasonable agreement with the value of nine α-CD units determined independently by quantitative ^{1}H NMR spectroscopy. GPC analysis of PR-4MA and PR-8MA showed a progressive increase in molecular weight accompanied by only a modest increase in PDI, supporting the successful grafting of PEGMA side

chains onto the polyrotaxane backbone. The relatively low PDI values further suggest limited variation in the average degree of PEGMA functionalization among different polyrotaxane molecules. The average number of grafted PEGMA side chains was estimated from the difference between the number-average molecular weights of PR-xMA and PR, divided by the average molecular weight of a single PEGMA chain (~500 Da). This analysis yielded approximately four (PR-4MA) and eight (PR-8MA) PEGMA substituents per polyrotaxane, in good agreement with the values independently determined by quantitative ^{1}H NMR spectroscopy.

2. Film Characterizations

2.1 Scanning Electron Microscopy

Cross-section images of polymer films were taken with SEM and are reported in Figure S6.

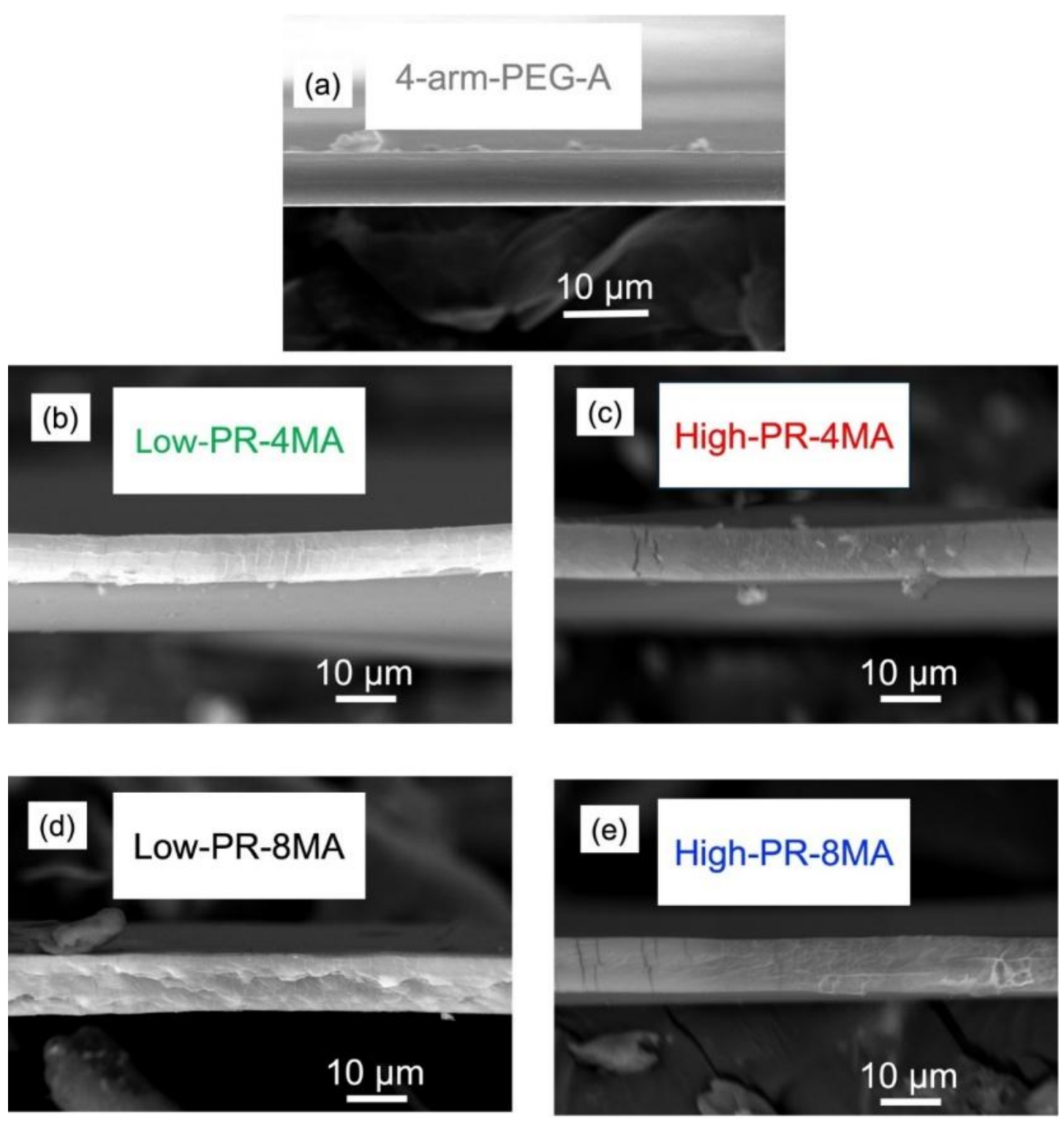

Figure S6. Cross-sectional SEM images of the polymer films: (a) 4-arm-PEG-A, (b) Low-PR-4MA, (c) High-PR-4MA, (d) Low-PR-8MA, and (e) High-PR-8MA.

Film thicknesses estimated from the images were 6.4±0.1 μm for the control film of 4-arm-PEG-A, 8.4±0.3 μm for Low-PR-4MA, 9.9±0.3 μm for High-PR-4MA, 10.3±0.1 μm for Low-PR-8MA, and 9.6±0.3 μm for High-PR-8MA.

2.2 FT-IR Spectroscopy

The FTIR spectra of PR-4MA, PR-8MA, 4-arm-PEG-A, α-cyclodextrin (α-CD), and the polymer films obtained with different PR-xMA:4-arm-PEG-A ratios, following photopolymerization and air-drying, are shown in Figure S7. Characteristic bands of both the PEG backbone (C–O stretching in the 1060-1150 cm^{-1} region)[4] and α-CD (C–O/C–C stretching at 1030 cm^{-1} and ring vibration at 705 cm^{-1})[5] were observed in the spectra of all the polymer films, confirming the incorporation of both 4arm-PEG-A and PR components.

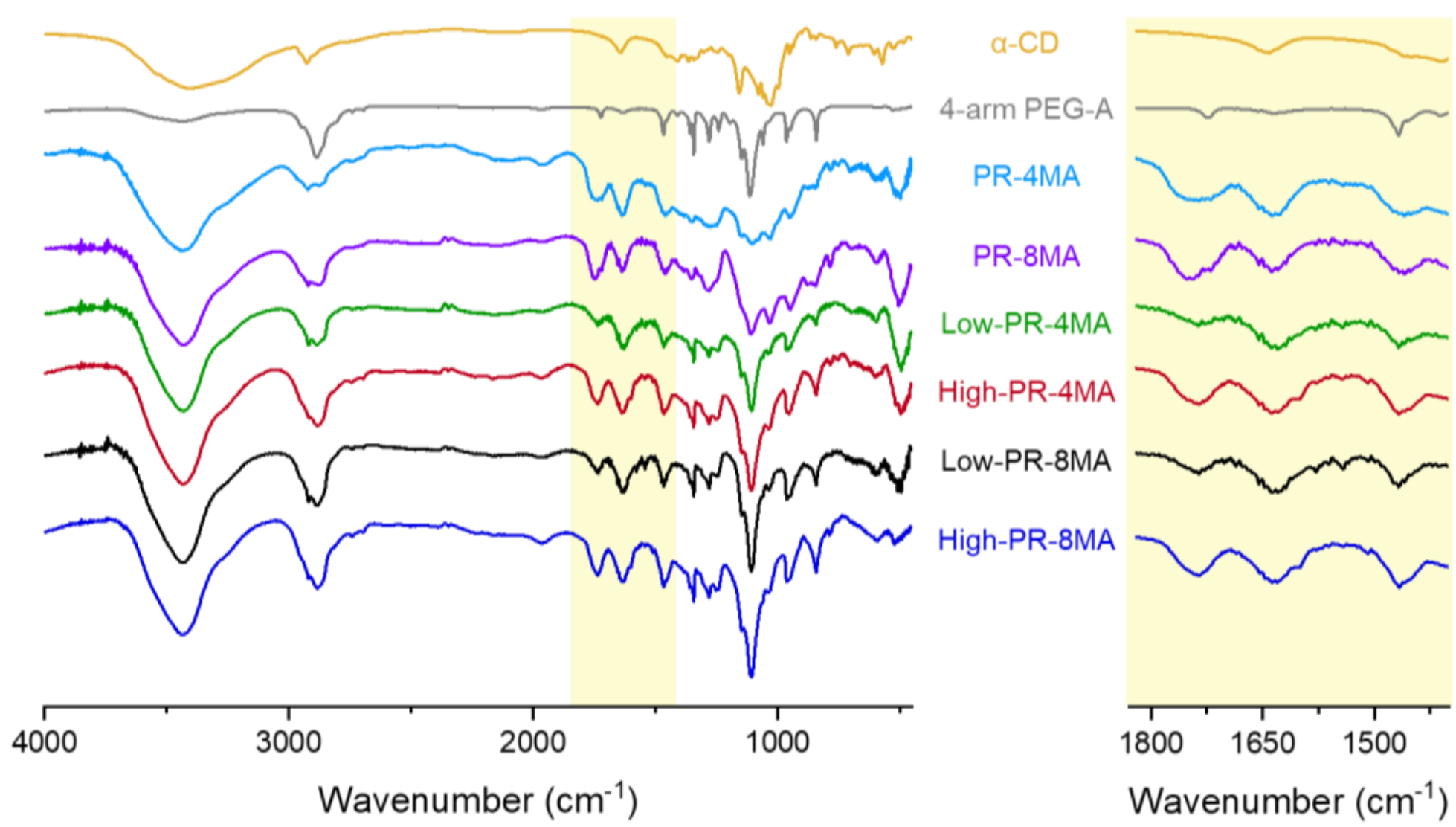

Figure S7. FTIR spectra of α-CD (gold), 4-arm-PEG-A (gray), and PR functionalized with low (light blue) and high (purple) densities of PEGMA side chains. Also shown are the spectra of films prepared with low (green) and high (red) ratios of PR-4MA to 4-arm-PEG-A, and with low (black) and high (dark blue) ratios of PR-8MA to 4-arm-PEG-A.

All films displayed a distinct band at 1746 cm^{-1}, corresponding to the stretching vibration of the carbonate group.[1] This band originates from the carbonate linker of the PEGMA side chains grafted to the α-CD rings, indicating that the PEGMA substituents remained covalently attached to the PR and were successfully crosslinked into the film matrix after photopolymerization. The bands at 3431 cm^{-1} characteristic of –OH stretching attributed to α-CDs ring, and the bands in the region 2800–3000 cm^{-1}, characteristic of –CH stretching, display similar intensities for all the films, and also when compared to the respective PR-4MA and PR-8MA monomers employed for polymerization, indicating that no significant amount of water remains incorporated into the films following polymerization and air-drying. In addition, all films exhibited a band at 1344 cm^{-1}, attributed to the bending vibration of $–CH_2$ groups in the PEG chains.

Notably, in films prepared with a low ratio of PR-xMA to 4-arm-PEG-A (Low-PR-4MA and Low-PR-8MA), the 1344 cm^{-1} band was significantly more intense than the 1746 cm^{-1} band, consistent with a higher content of 4-arm-PEG-A in the film composition. Conversely, in films prepared with a High-PR-xMA:4arm-PEG-A ratio (High-PR-4MA and High-PR-8MA), the intensities of the two bands became comparable, confirming the increased contribution of PR within the polymer networks.

2.3 Determination of Polymer Films Composition

The composition of the polymer films was determined by quantitative ^1H NMR spectroscopy following alkaline hydrolysis of the films and neutralization of excess alkali. The experimental composition (EC), expressed as the ratio between proton contributions from cyclodextrins (α-CD) and PEG, was compared to the theoretical composition (TC) calculated from the monomer feed ratios. The ^1H NMR spectrum of α-CD in D_2O (Figure S8a) shows two distinct sets of signals: a

doublet at δ 5.30, attributed to the 6 anomeric (C_1-H) protons, and two multiplets at δ 4.30–3.75, attributed to the remaining 36 protons of the glucose units. The spectrum of dissolved 4-arm-PEG-A film (Figure S8b) displays multiple resonances in the region δ 2.60–1.60, characteristic of polymerized acrylate protons, and a broad signal in the region δ 4.57–3.45 arising from PEG protons, with no signals observed in the region δ 5.30 corresponding to the proton bonded to C_1 of α-CD. The acrylate proton signal, corresponding to 16 protons per polymerized PEG-A molecule, was used as an internal reference because it does not overlap with other resonances, and leads to an estimation of 945 protons per 4-arm-PEG-A, in good agreement with the previous integral observed for PEG (MW 10 kDa) (see Figure S1).

The experimental composition (EC) was determined from the ratio between the integral of the signals in the region δ 4.57–3.45, arising from both PEG and α-CD protons, and the integral of the α-CD anomeric protons at δ 5.30, which was used as the internal reference and normalized to 1. ECs were then compared to the theoretical values (TC) estimated according to the different feed ratios, determined using the following equation:

$$TC = \frac{945(3.75 + n) + 46(X * n) + 36(9 * n)}{6(9 * n)}$$

where 945 represents the theoretical protons provided by PEG (MW 10 kDa); 3.75 is the μmol of 4-arm-PEG-A and n is the μmol of PR-xMA employed for polymerizations; 46 represents the theoretical protons provided by PEGMA (MW 500 Da) and X is the grafting degree of PR-xMA; 36 represents the theoretical protons provided by α-CD in the region δ 4.57–3.45; 9 represents the number of α-CDs per PR-xMA unit; 6 represents the theoretical protons provided by α-CD in the region δ 5.30.

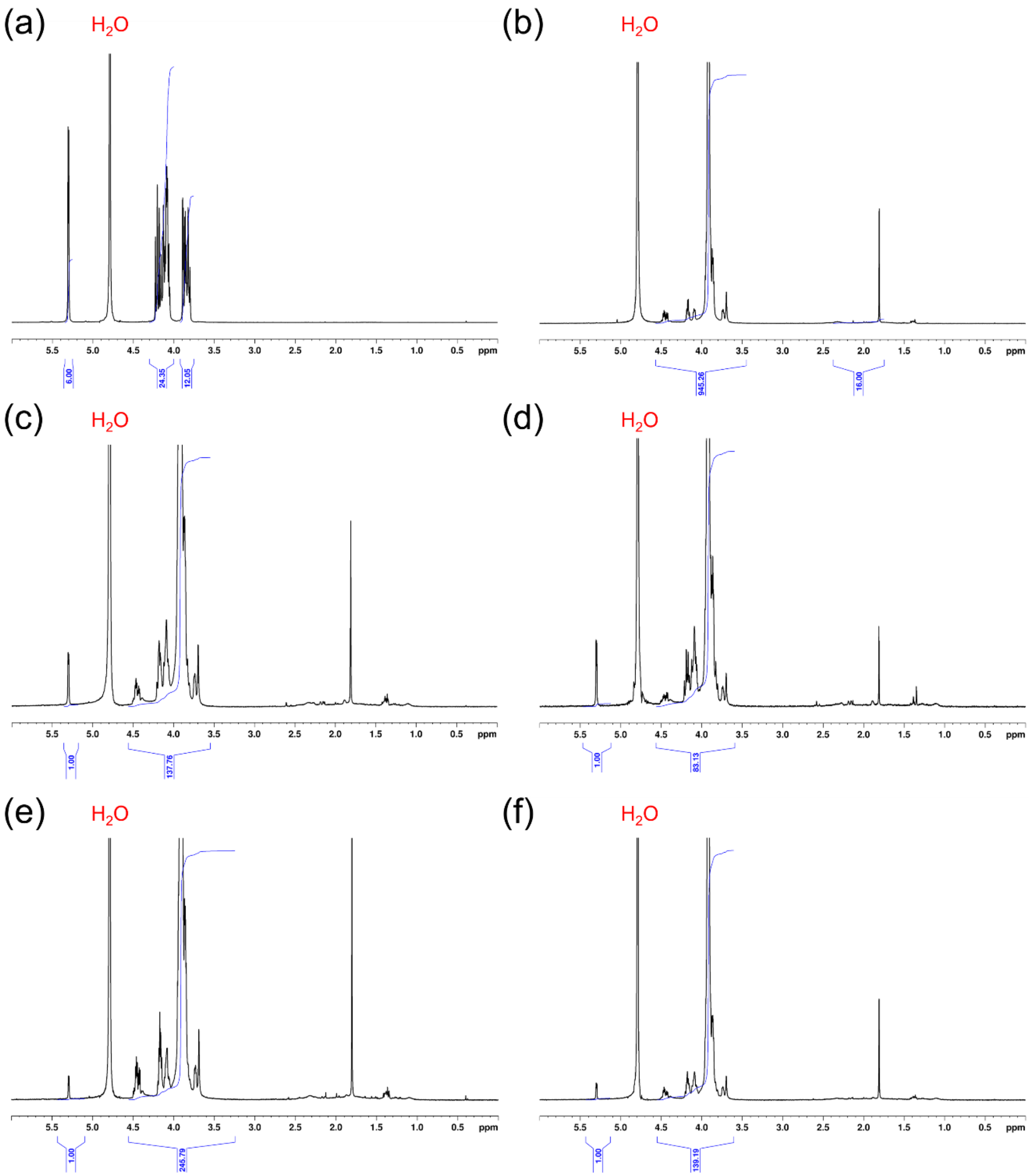

Figure S8. ^{1}H NMR spectra (400 MHz, D_2O, 298 K) of (a) α-CD and of dissolved polymer films through alkaline hydrolysis: (b) 4-arm-PEG-A, (c) Low PR-4MA, (d) High PR-4MA, (e) Low PR-8MA and (f) High PR-8MA.

Under these conditions, films prepared with Low-PR-4MA (0.61 μmol PR) and High-PR-4MA (1.22 μmol PR) exhibited EC values of 138:1 (Figure S8c) and 83:1 (Figure S8d), respectively, in good agreement with the corresponding TC values of 136:1 and 80:1. Similarly, films prepared with Low-PR-8MA (0.31 μmol PR) and High-PR-8MA (0.61 μmol PR) exhibited EC values of 246:1 (Figure S8e) and 139:1 (Figure S8f), in close agreement with the corresponding TC values of 245:1 and 137:1, respectively. These results demonstrate that the compositions of the crosslinked films closely match the monomer feed ratios, indicating quantitative incorporation of both PR-xMA and 4-arm-PEG-A during polymerization.

2.4 Mechanical Characterization

Tensile tests were performed using a custom-made platform composed of a piezoelectric actuator and a calibrated spring load sensor mounted on a silicon substrate (Figure S9), adapted from a platform previously reported by us.[6]

Thin film specimens were cut from as-prepared films using a stainless-steel blade into a rectangular shape (length ~2.5-3.5 mm, width ~1-2 mm) and pasted onto the two silicon slices at opposite ends, whose distance corresponded to the specimen's gauge length. During the tensile test, the actuator delivered a displacement to the silicon slice connected to it, causing the specimen to stretch and the consecutive deformation of the calibrated spring. According to this configuration, the calibrated spring and the specimen behave like a series of springs and thus experience the same load. Images of the two silicon slices supporting the specimen were recorded and then analyzed by custom-made image correlation software implemented in C language, in order to evaluate the displacement of the specimen's opposite ends. All the tests were performed at room temperature.

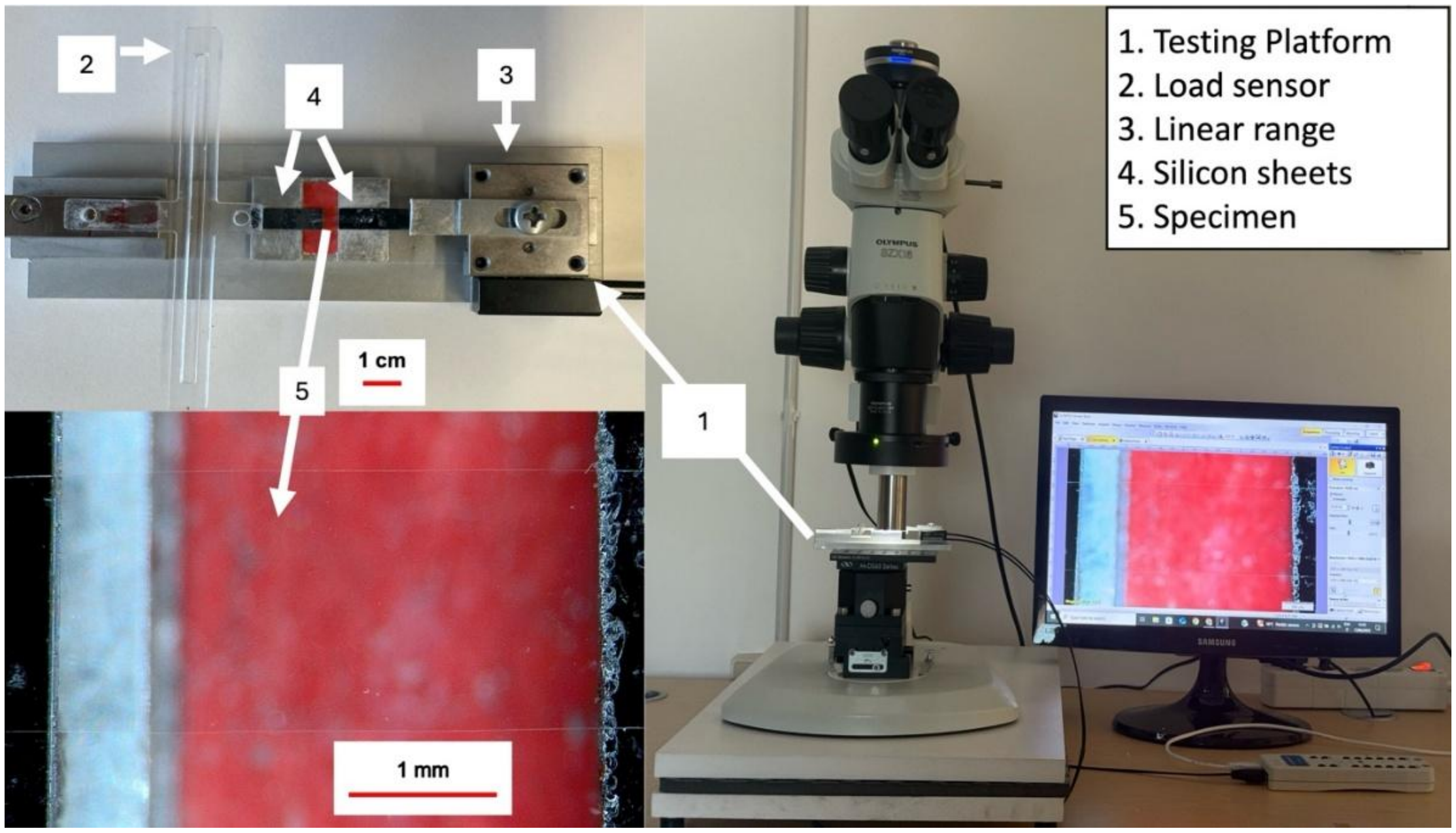

Figure S9. Experimental setup for the uniaxial tensile test of PR-based films.

The strain was determined as the ratio of the relative displacement of the specimen's ends and its undeformed gauge length. Force was obtained from the product of the spring constant of the load sensor and its displacement. Force divided by the cross-sectional area of the film allowed for the nominal stress to be obtained. The elastic modulus was calculated as the slope of the stress-strain curves in the initial region. The toughness was obtained from the area under the stress-strain curves.

Figure S10 shows the stress-strain curves for specimens obtained from the films at different PR-xMA:4-arm-PEG-acrylate ratios. In particular, Figure S10a displays the curves from films based on PR-4MA, while Figure S10b displays the curves from films based on PR-8MA. The mechanical properties reported for polyrotaxane-based films in Table 1 were obtained from the average of three specimens of each film.

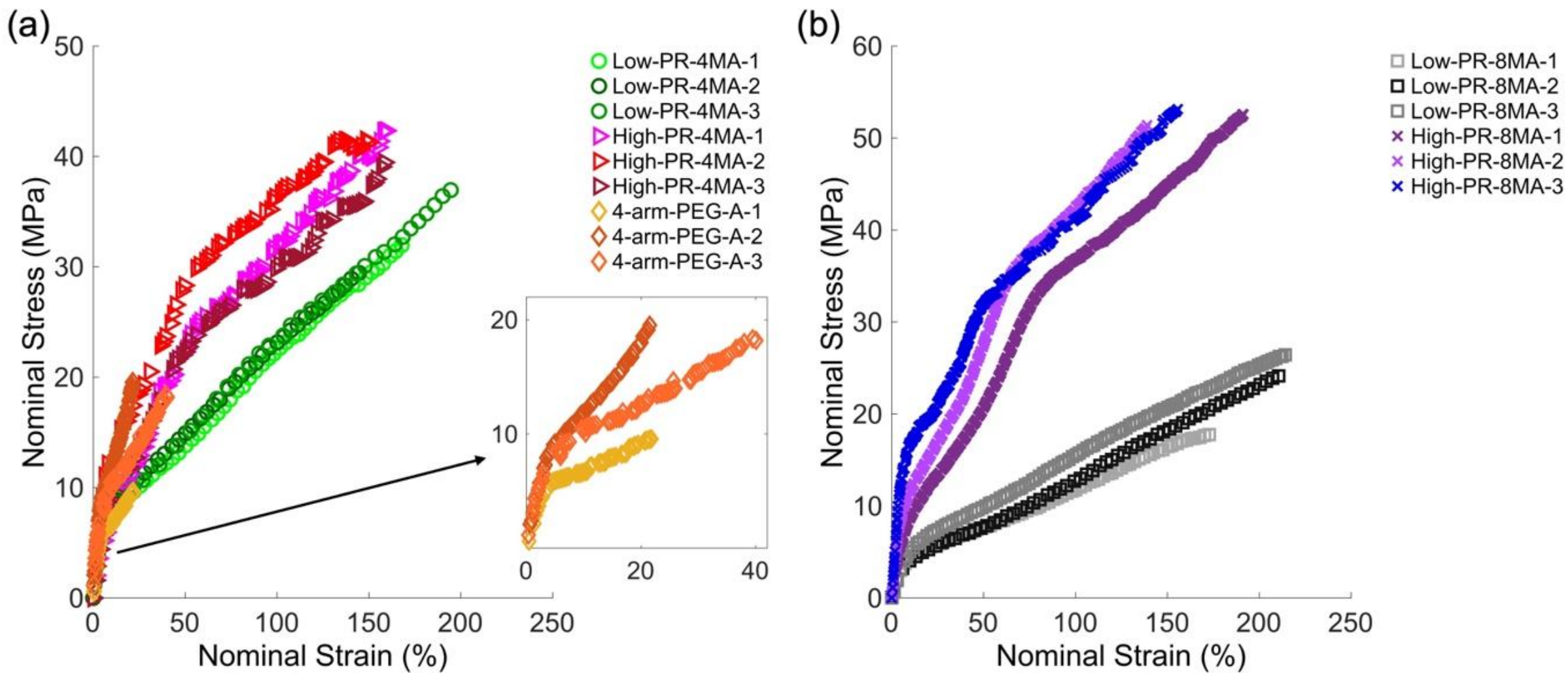

Figure S10. Stress-strain curves derived from uniaxial tensile tests of polymer thin films of (a) 4-arm-PEG-A (control sample) and different ratios of (a) PR-4MA and (b) PR-8MA.

To understand the variation in the slope of the stress-strain curves, normalized stiffness was plotted against strain, and the results are shown in Figure S11. The normalized stiffness at any strain value is determined by the slope of the stress-strain curve at the corresponding strain value divided by the maximum elastic modulus among the films (i.e., slope of the stress-strain curve at the initial region of High-PR-8MA).[7] In the initial strain range (0-30%), all the films exhibit similar behavior, with a decrease in stiffness (softening) with increasing strain. In the 30-70% strain region, an interesting increase in stiffness is observed for High-PR-4MA and High-PR-8MA films, followed by a decrease. This variation of stiffness with strain appeared as a kink in the stress-strain curve and could be related to the content of PR, which is higher in these films.

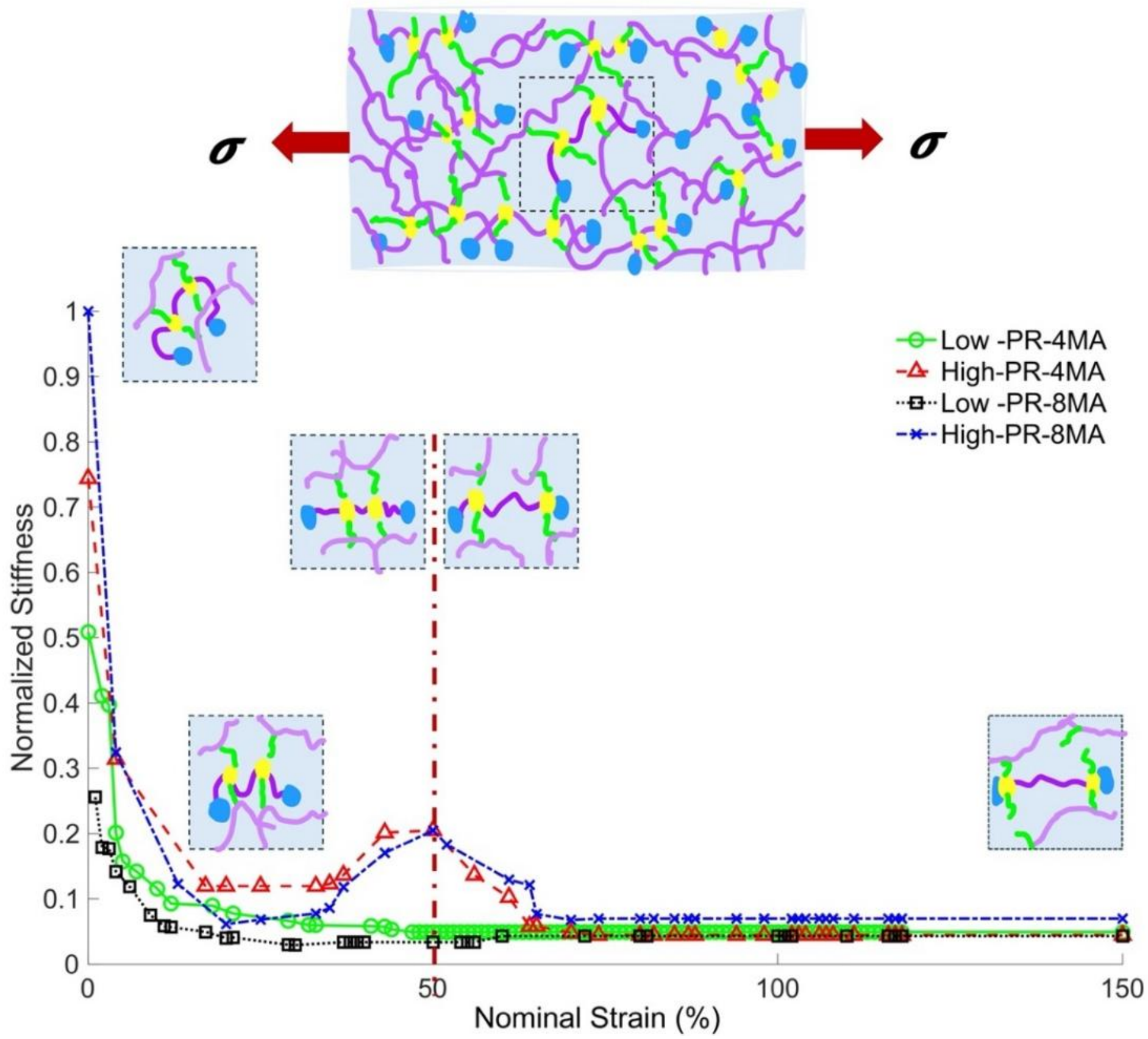

Figure S11. Variation of normalized stiffness (computed as the slope of the stress-strain curve divided by the maximum stiffness value at the initial region of High-PR-8MA film) of PR-based films with the applied strain.

Figure S12 illustrates the response of as-prepared PR-films against cyclic loading for different applied strain amplitudes, respectively, 20%, 70%, and 100%.

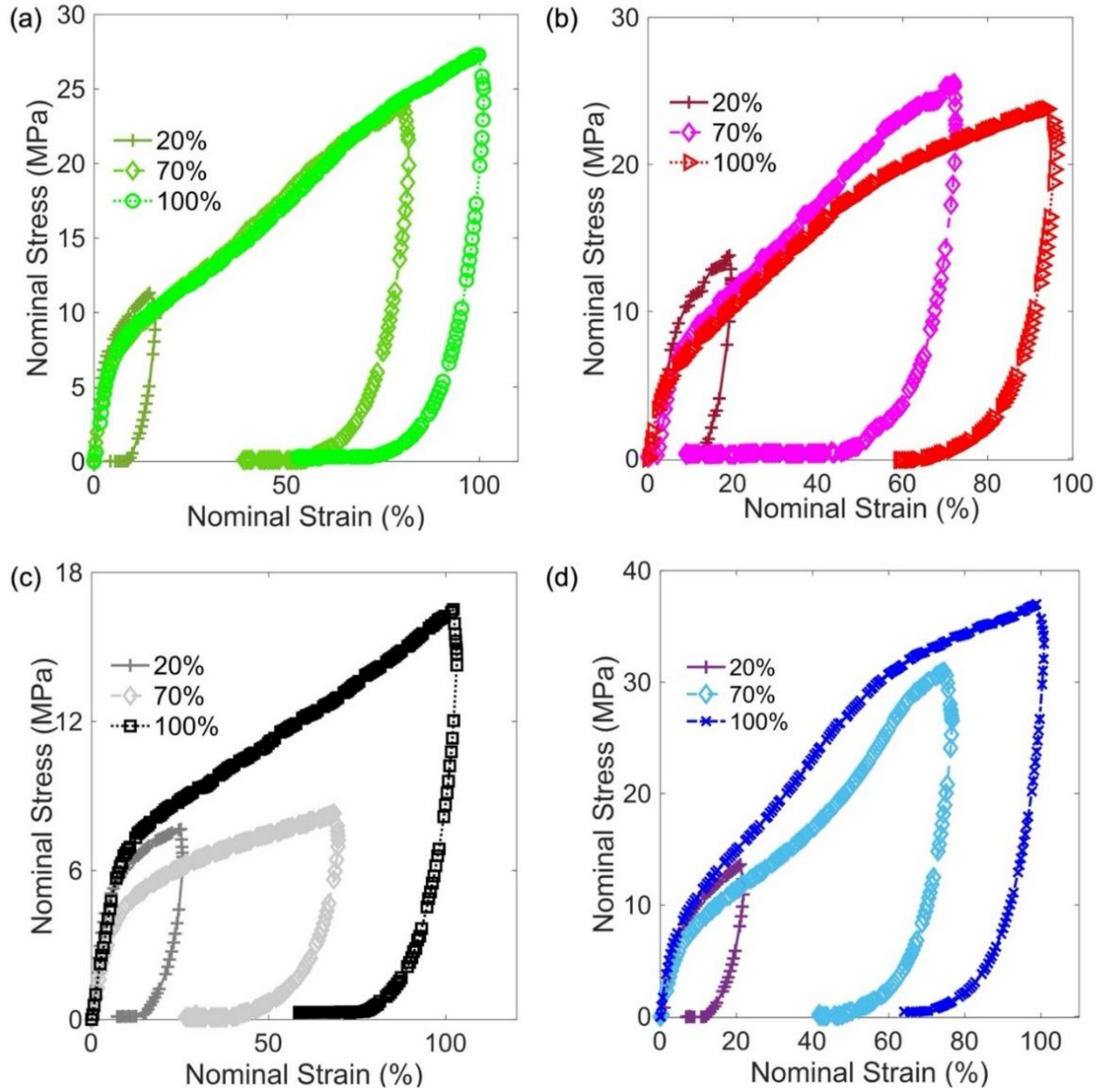

Figure S12. Cyclic loading-unloading response at 20, 70, and 100% applied strain of the PR-based thin films: (a) Low-PR-4MA, (b) High-PR-4MA, (c) Low-PR-8MA, and (d) High-PR-8MA.

Figure S13 shows the response of the films under the application of consecutive cycles of loading and unloading. After the first cycle, all the consecutive curves are similar, demonstrating the mechanical robustness of the developed films under consecutive deformations.

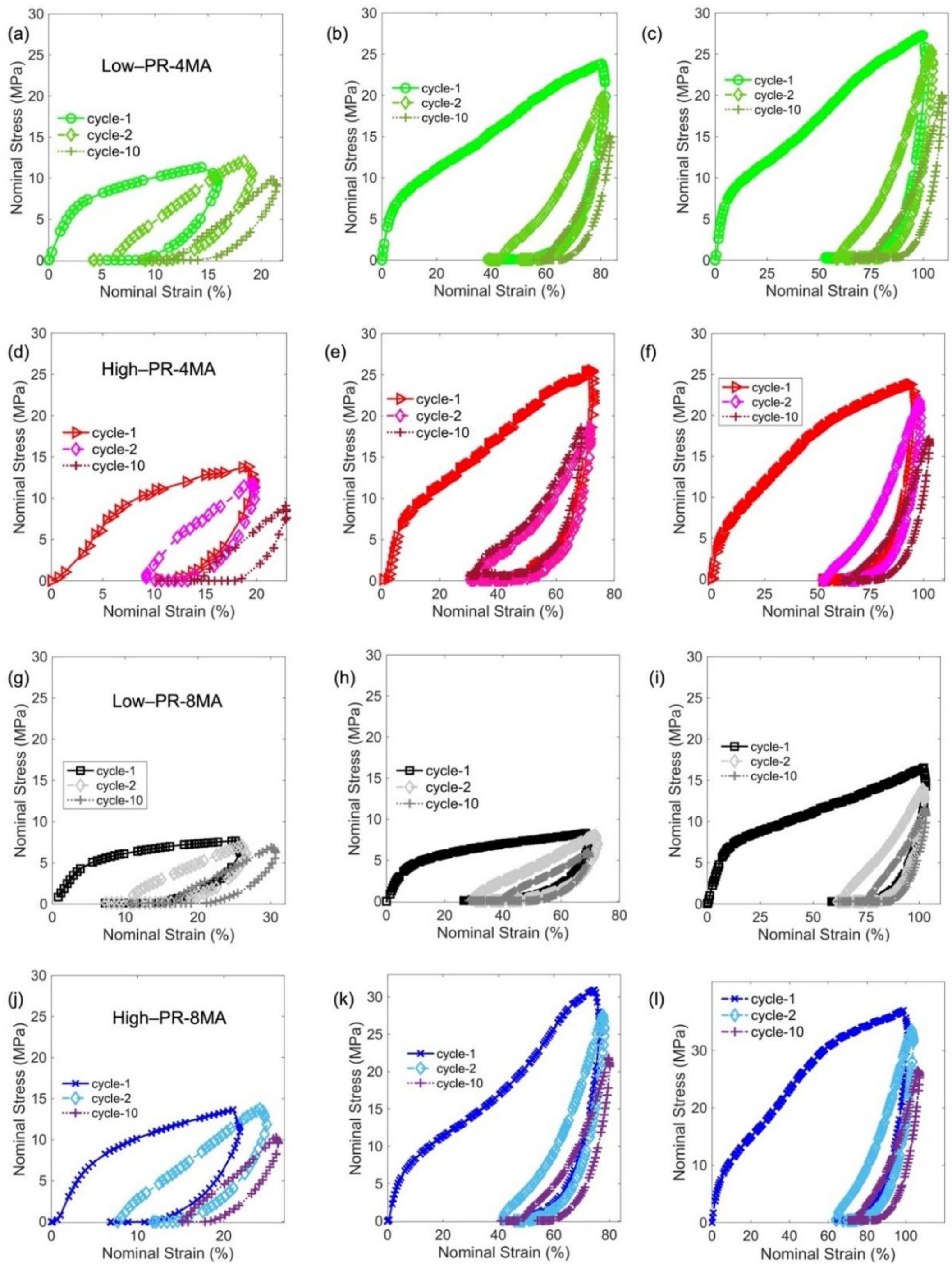

Figure S13. First, second, and tenth cyclic loading-unloading response of Low-PR-4MA (a, b, c), High-PR-4MA (d, e, f), Low-PR-8MA (g, h, i), and High-PR-8MA (j, k, l) films subjected to different applied strain values, respectively 20%, 70%, and 100%.

2.5 Thermal Analysis

The thermal properties of the polymer films were evaluated by differential scanning calorimetry analysis. Samples of ~5 mg were tested from 50 °C to 300 °C at a rate of 10 °C/min, and the results are reported in Figure S14.

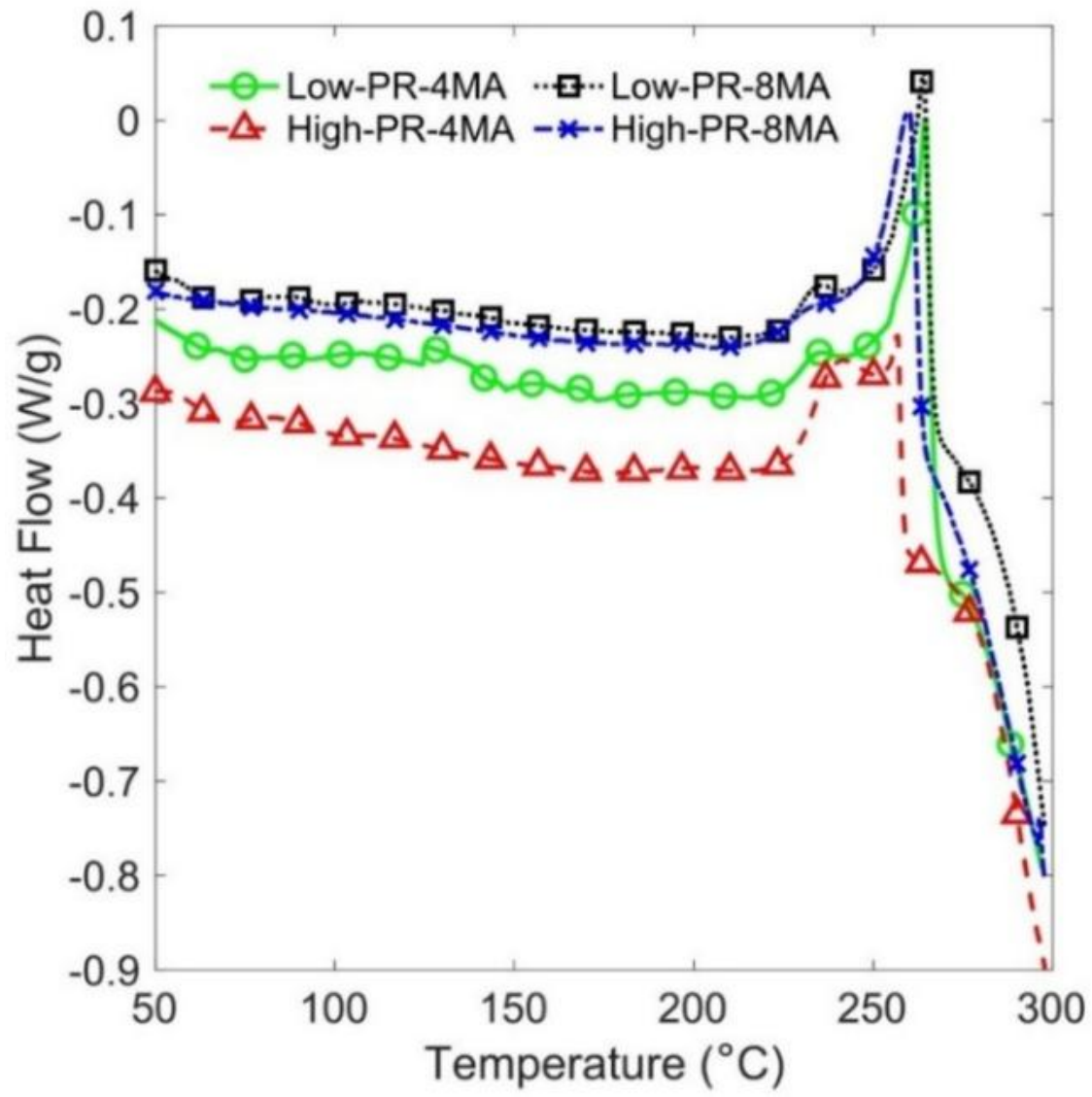

Figure S14. Heat flow curves obtained from DSC analysis of PR-xMA films.

3. Temperature Sensor Fabrication

3.1 Sensors Fabrication

The fabricated temperature sensors with identical meander design produced by silver ink printing and masked CNT drop-casting, featuring 450 μm line width, 1.05 mm pitch, $1.125 \times 1.125\ mm^2$ square pads, a pad-to-pad distance of 17 mm, and a total length of 71.625 mm, are shown in Figure S15.

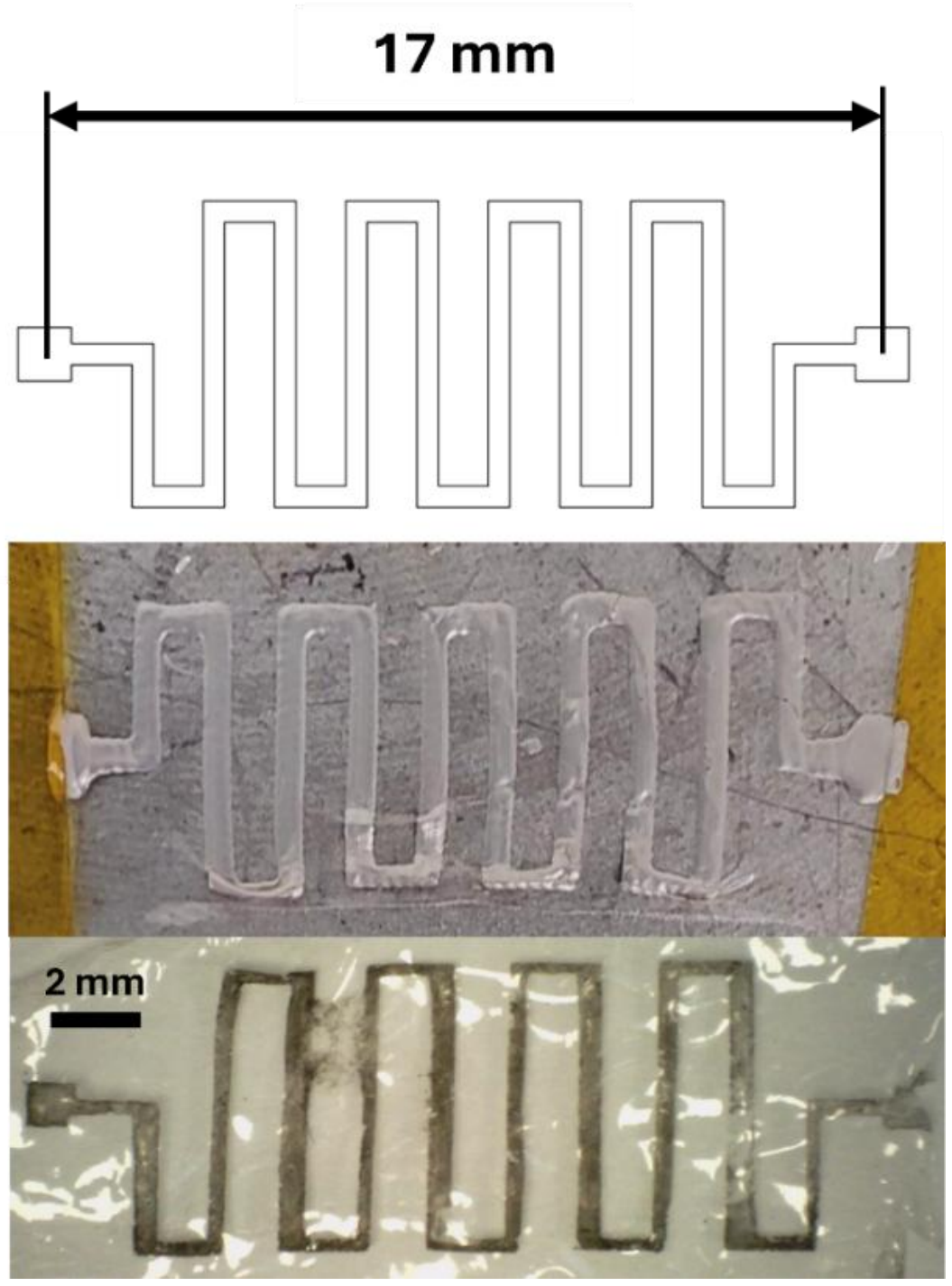

Figure S15. Pictures of the layout for the temperature sensor (top), and the obtained layouts on High-PR-8MA film by ink printing using Ag ink (middle), and the drop-casting process from 0.05% w/w CNT aqueous solution (bottom).

3.2 Sensors Investigation

I–V curves for the CNT-based sensor, collected at different temperatures, are shown in Figure S16a. A progressive increase in current is observed at higher temperatures, consistent with the negative temperature coefficient of resistance of CNT-based conductive materials. After cooling the CNT-based sensor back to 25 °C, it was subjected to ~60% uniaxial strain in the pad-to-pad direction. I–V curves were recorded under strain application at increasing temperatures, every 5 °C between 25 °C and 75 °C, and reported in Figure S16b. When compared to the unstretched film, the overall current recorded at the same temperature decreases in the stretched sensor, indicating a

higher resistance associated with elongation of the conductive pathways. However, the progressive increase in current with temperature can also be observed under strain.

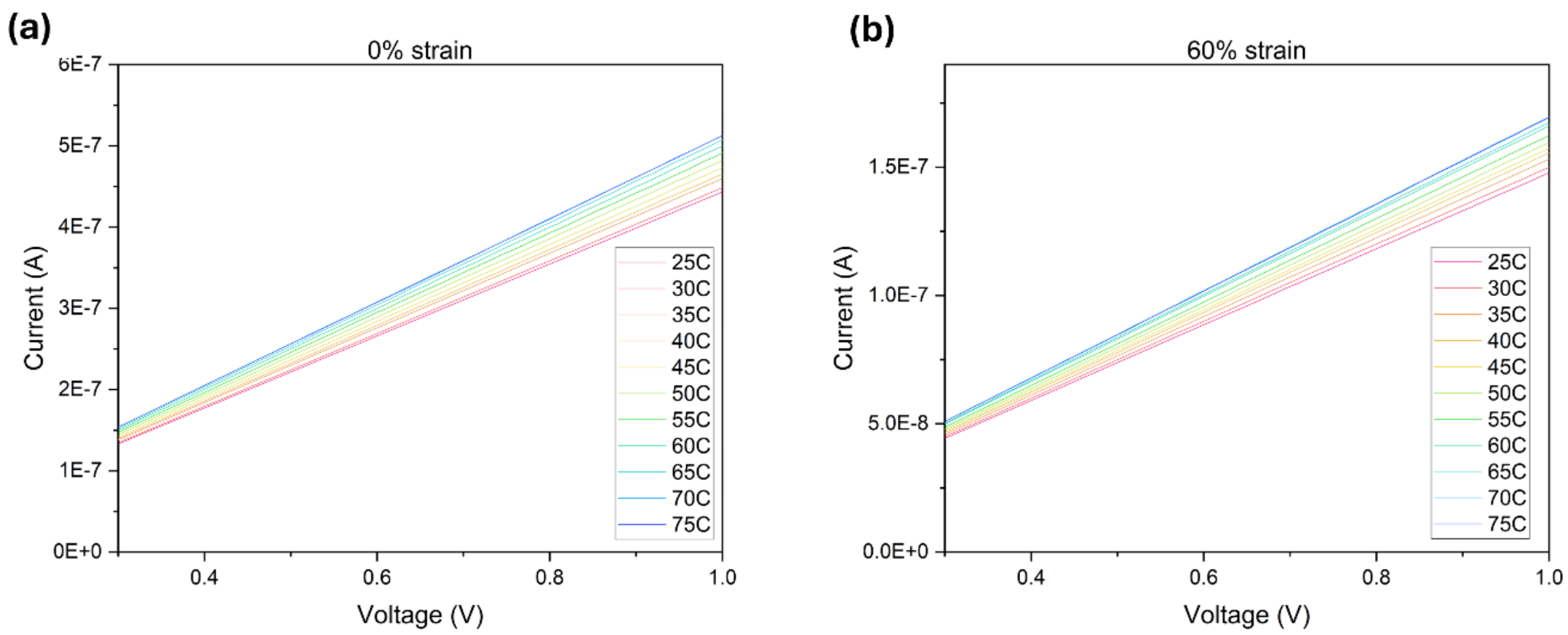

Figure S16. I–V curves, in the 0.3–1V range used for resistance calculations, of the CNT-based temperature sensor under the application of (a) 0% and (b) 60% strain.

Figure S17a displays the experimental setup for the investigation of the unstretched CNT-based sensor, and Figure S17b shows the sensor under ~60% strain. The strained sensor was secured in place using Kapton tape to maintain a constant applied deformation during the measurement.

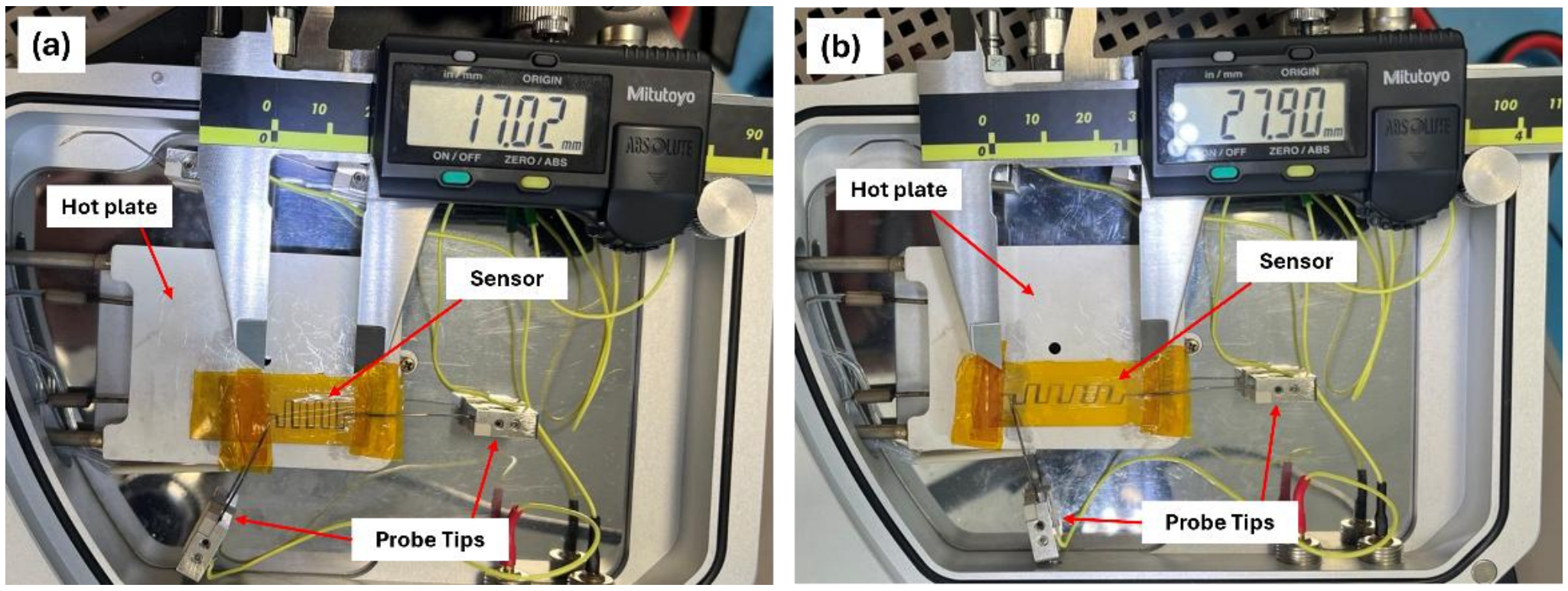

Figure S17. Experimental setup for the characterization of the CNT-based temperature sensor (a) as prepared and (b) after the application of ~60% strain.

4. References

(1) Jiang, Y.; Zhang, Z.; Wang, Y. X.; Li, D.; Coen, C. T.; Hwaun, E.; Chen, G.; Wu, H. C.; Zhong, D.; Niu, S.; Wang, W.; Saberi, A.; Lai, J. C.; Wu, Y.; Wang, Y.; Trotsyuk, A. A.; Loh, K. Y.; Shih, C. C.; Xu, W.; Liang, K.; Zhang, K.; Bai, Y.; Gurusankar, G.; Hu, W.; Jia, W.; Cheng, Z.; Dauskardt, R. H.; Gurtner, G. C.; Tok, J. B. H.; Deisseroth, K.; Soltesz, I.; Bao, Z. Topological Supramolecular Network Enabled High-Conductivity, Stretchable Organic Bioelectronics. *Science* **2022**, *375*, 1411–1417. https://doi.org/10.1126/science.abj7564.

(2) Harada, A.; Li, J.; Kamachi, M. The Molecular Necklace: A Rotaxane Containing Many Threaded α-Cyclodextrins. *Nature* **1992**, *356* (6367), 325–327. https://doi.org/10.1038/356325A0;KWRD.

(3) Schneider, H. J.; Hacket, F.; Rüdiger, V.; Ikeda, H. NMR Studies of Cyclodextrins and Cyclodextrin Complexes. *Chem. Rev.* **1998**, *98* (5), 1755–1785. https://doi.org/10.1021/CR970019T.

(4) Matsuura, H.; Miyazawa, T.; Machida, K. Infrared Spectra of Poly(Ethylene Glycol) Dimethyl Ethers in the Crystalline State. *Spectrochim. Acta Part A* **1973**, *29* (5), 771–779. https://doi.org/10.1016/0584-8539(73)80044-3.

(5) Casu, B.; Reggiani, M. Infrared Spectra of Amylose and Its Oligomers. *J. Polym. Sci. Part C: Polym. Symp.* **1964**, *7* (1), 171–185. https://doi.org/10.1002/POLC.5070070113.

(6) Missale, E.; Maniglio, D.; Speranza, G.; Frasconi, M.; Pantano, M. F. Cellulose Nanocrystal Composites with Enhanced Mechanical Properties for Robust Transparent Thin Films. *ACS Appl. Nano Mater.* **2024**, *7*, 18167–18176. https://doi.org/10.1021/acsanm.3c02946.

(7) Manish, V.; Arockiarajan, A.; Tamadapu, G. Influence of Water Content on the Mechanical Behavior of Gelatin Based Hydrogels: Synthesis, Characterization, and Modeling. *Int. J. Solids Struct.* **2021**, *233*, 111219. https://doi.org/10.1016/J.IJSOLSTR.2021.111219.
